# Unraveling the genetic tapestry of pediatric sarcomeric cardiomyopathies and masquerading phenocopies in Jordan

**DOI:** 10.1038/s41598-024-64921-9

**Published:** 2024-07-02

**Authors:** Bilal Azab, Dunia Aburizeg, Sherin T. Shaaban, Weizhen Ji, Lina Mustafa, Nooredeen Jamal Isbeih, Amal Saleh Al-Akily, Hashim Mohammad, Lauren Jeffries, Mustafa Khokha, Saquib A. Lakhani, Iyad Al-Ammouri

**Affiliations:** 1 Division of Pathology and Laboratory Medicine, Phoenix Children’s Hospital, Phoenix, AZ 85016 USA; 2 Department of Pathology and Microbiology and Forensic Medicine, School of Medicine, The University of Jordan, Amman, 11942 Jordan; 3 Department of Biology and Biotechnology, Faculty of Science, American University of Madaba, Madaba, 11821 Jordan; 4 Department of Pediatrics, Pediatric Genomics Discovery Program, Yale University School of Medicine, New Haven, CT 06510 USA; 5 Department of Genetics, Yale University School of Medicine, New Haven, CT 06510 USA; 6 Department of Pediatrics, School of Medicine, The University of Jordan, Amman, 11942 Jordan

**Keywords:** DNA, Genetics research, Paediatric research, Clinical genetics, Disease genetics, Genetic testing, Genetics, Cardiology, Molecular medicine

## Abstract

Pediatric cardiomyopathies are mostly attributed to variants in sarcomere-related genes. Unfortunately, the genetic architecture of pediatric cardiomyopathies has never been previously studied in Jordan. We sought to uncover the genetic landscape of 14 patients from nine families with several subtypes of pediatric cardiomyopathies in Jordan using Exome sequencing (ES). Our investigation identified pathogenic and likely pathogenic variants in seven out of nine families (77.8%), clustering in sarcomere-related genes. Surprisingly, phenocopies of sarcomere-related hypertrophic cardiomyopathies were evident in probands with glycogen storage disorder and mitochondrial-related disease. Our study underscored the significance of streamlining ES or expanding cardiomyopathy-related gene panels to identify plausible phenocopies of sarcomere-related cardiomyopathies. Our findings also pointed out the need for genetic testing in patients with cardiomyopathy and their at-risk family members. This can potentially lead to better management strategies, enabling early interventions, and ultimately enhancing their prognosis. Finally, our findings provide an initial contribution to the currently absent knowledge about the molecular underpinnings of cardiomyopathies in Jordan.

## Introduction

Pediatric cardiomyopathy (CMP) is a group of rare heterogeneous diseases, estimated to affect around 1 in 100,000 children under 18, with a significantly higher incidence during the first two years of life^1–4^. CMPs are characterized by structural, mechanical, and/or electric dysfunction of the myocardium and are attributed to a variety of causes that are frequently genetic^5^. CMPs can either be confined to the heart or be a part of a systemic disorder. There are five subtypes of CMP: hypertrophic (HCM), restrictive (RCM), dilated (DCM), arrhythmogenic right ventricular (ARVC), and left-ventricular non-compaction (LVNC)^5^. HCM and DCM are the most common types, whereas LVNC and RCM are less frequent, and ARVC is rarely diagnosed in childhood^2,4^.

More than 100 genes have been implicated in causing pediatric CMP, encoding Z-band, nuclear membrane, desmosomal, mitochondrial, cytoskeletal, intracellular calcium modulator, sarcomere, and sarcomere-associated binding proteins, among others^6,7^. Several studies showed that pathogenic variants in such genes can manifest with variable expressivity and incomplete penetrance in 26–39% of pediatric-onset CMP patients^8,9^. Interestingly, some syndromes can present with cardiomyopathies mimicking sarcomeric HCM but stemming from distinct genetic origins, termed HCM phenocopies^10^. For instance, lysosomal storage, mitochondrial, and glycogen storage disorders can be identified as HCM phenocopies^10^. Differential diagnosis between sarcomeric HCM and HCM phenocopies can significantly impact management and prognosis^10^.

Genetic testing is therefore of great value in cardiomyopathies, especially in familial cases. By identifying putative pathogenic variants in probands, a cascade of genetic testing for at-risk first-degree family members can be triggered^11^ to help improve the management plan for patients and their at-risk family members^10^. Currently, there are a variety of genetic tests available for cardiomyopathies, ranging from cardiomyopathy-targeted panels to more comprehensive approaches like -exome and genome sequencing. Advances in sequencing technologies have made genetic tests faster and more affordable, resulting in increased global adoption of these tests^11^.

Unfortunately, the genetic architecture of CMPs is under-investigated in the Middle East, increasing the likelihood of inconclusive findings in patients with CMPs from this region^12–14^. To our knowledge, the genetic basis of pediatric CMPs has never been previously studied in Jordan. In this study, we aimed to identify the molecular etiologies of pediatric cardiomyopathies in nine Jordanian families by utilizing ES. Furthermore, we aimed to emphasize the significance of expanded genetic testing in Jordan, as a genetically under-representative population, to proactively monitor and introduce management plans for patients with cardiomyopathies and their at-risk relatives.

## Results

### Overview

**Figure 1:**
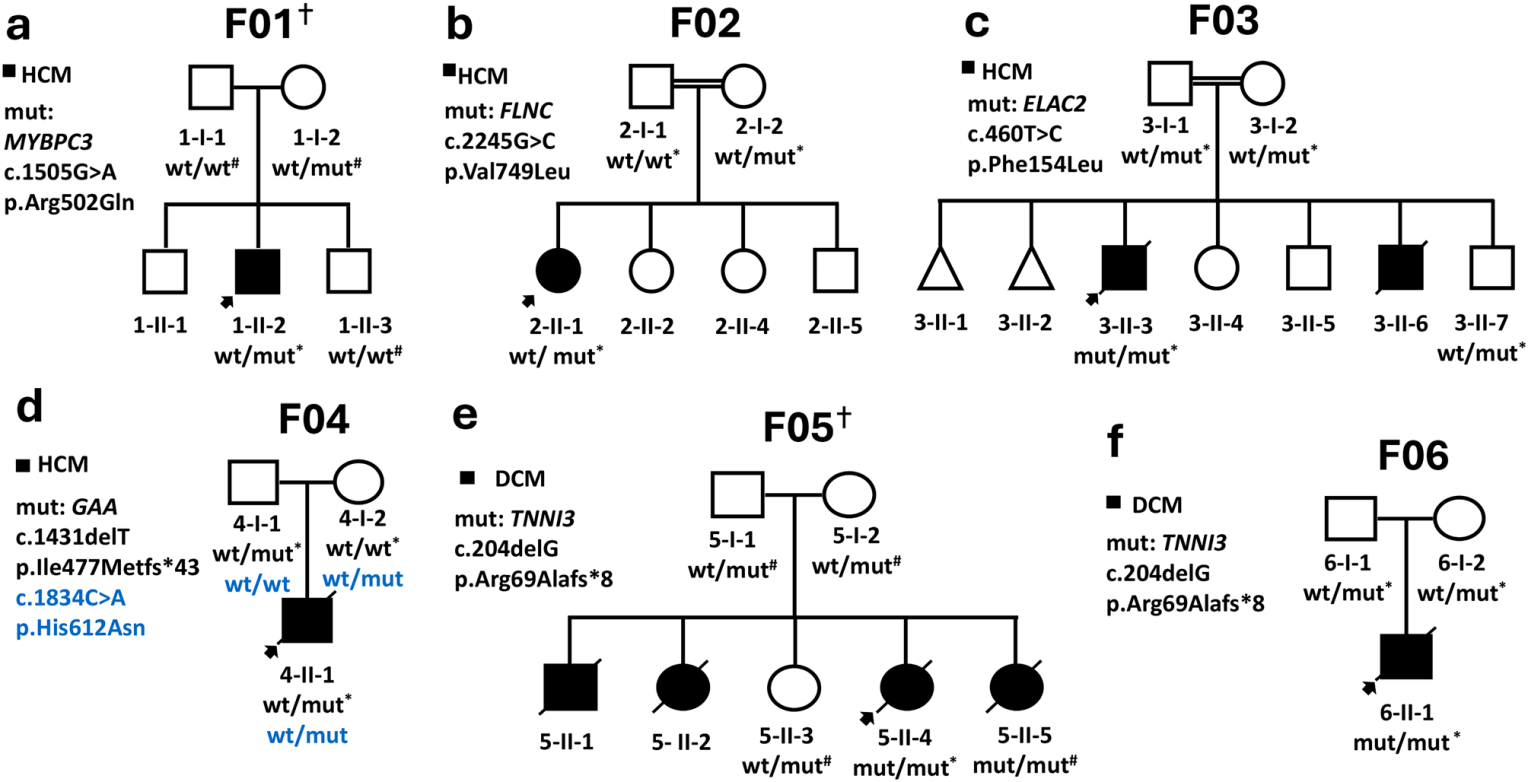
Pedigrees for families (F01–F06) affected by cardiomyopathy. *Samples that underwent ES. ^#^Samples sequenced by Sanger. ^†^Exome aligned to hg38, while the remaining exomes were aligned to hg19.

**Figure 2:**
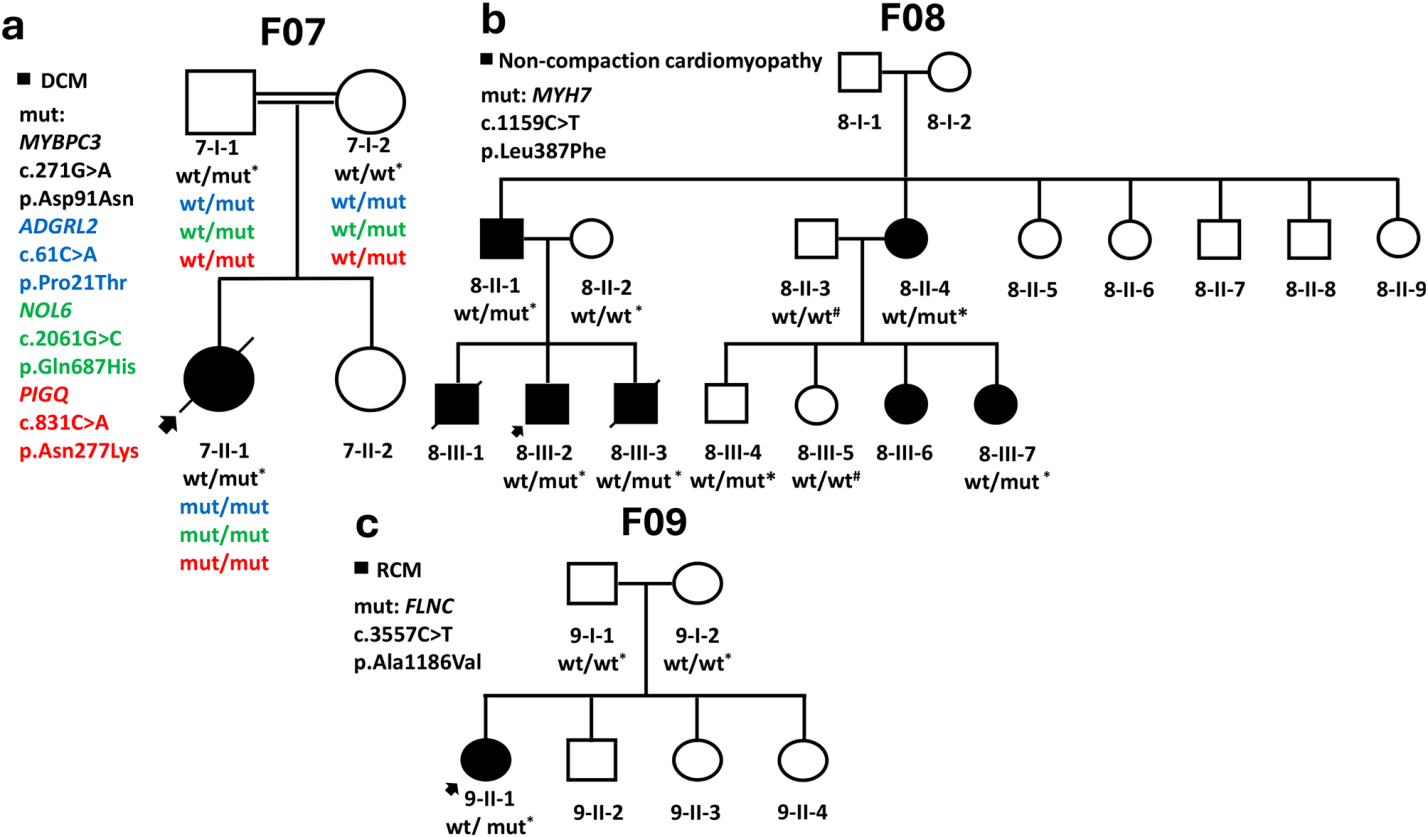
Pedigrees for families (F07–F09) with a history of cardiomyopathy. *Samples that underwent ES. ^#^Samples sequenced by Sanger. Those Exomes are aligned to hg19.

We recruited a cohort of 14 patients from nine families with either sporadic or familial cases presenting various subtypes of pediatric CMP. Specifically, four families with HCM (F01, F02, F03, and F04), three families with DCM (F05, F06, and F07), one family with LVNC (F08), and one family with RCM (F09) (Figs. 1, 2). The probands comprised five males and four females, with a median age at diagnosis of 6 months (range 0–8 years). The clinical characteristics of the probands are presented in Table 1.

**Table 1:** Clinical characteristics of the recruited probands.

Proband	Gene(s)	Sex	Age at diagnosis	Age of mortality	Condition	Presentation	Affected relatives (number)	Co-morbidities
1-II-2	*MYBPC3*	Male	7 years	Alive	HCM	Exertional chest pain Dizziness	No	Acute lymphocytic leukemia (in remission)
2-II-1	*FLNC*	Female	22 days	Alive	HCM	Feeding difficulty Respiratory distress	No	No
3-II-3	*ELAC2*	Male	6 months	7 months	HCM	Feeding difficulty Failure to gain weight Respiratory distress	Yes (1)	No
4-II-1	*GAA*	Male	2 months	4 months	HCM/Pompe	Feeding difficulty Tachypnea Dyspnea	No	Bilateral club foot
5-II-4	*TNNI3*	Female	9 months	18 months	DCM	Tachypnea Feeding difficulty Respiratory distress	Yes (3)	No
6-II-1	*TNNI3*	Male	10 months	13 months	DCM	Respiratory distress Progressive fatigue	No	No
7-II-1	*MYBPC3* and others	Female	2 years	3.5 years	DCM	Exercise intolerance	No	Autistic spectrum disorder Mild developmental delay
8-II-2	*MYH7*	Male	At birth	Alive	LVNC	Tachypnea Feeding difficulty Respiratory distress	Yes (6)	No
9-II-1	*FLNC*	Female	8 years	Alive	RCM	Progressive dyspnea Exercise intolerance Palpitation	No	No

*HCM* hypertrophic cardiomyopathy, *DCM* dilated cardiomyopathy, *LVNC* left ventricular noncompaction, *RCM* restrictive cardiomyopathy.

To uncover the molecular underpinnings of the patients’ cardiomyopathic presentation, we performed Exome sequencing (ES) analysis on selected members from each family (Figs. 1, 2). Plausible (likely) pathogenic variants and VUSs were identified in nine different genes, with variants in three genes, *MYBPC3*, *FLNC*, and *TNNI3*, appearing in two families each (Table 2). Eight of the nine families had (likely) pathogenic variants and VUS in a single gene, while family F07 had VUSs in four candidate genes. Notably, four of the candidate genes encoded sarcomere-related proteins (*TNNI3*, *MYBPC3*, *MYH7,* and *FLNC).* Two of the implicated genes (*ELAC2* and *GAA*) were associated with phenocopies of HCM, while the remaining variants were identified in genes of uncertain significance (*ADGRL2*, *NOL6*, and *PIGQ*) (Fig. S1). Overall, according to the ACMG guidelines^15^, six of the candidate variants were classified as variants of uncertain significance (VUS), two as likely pathogenic, and four as pathogenic (Table 2, Tables S1–S11, Figs. S1–S10).

**Table 2:** Candidate cardiomyopathy-causing variants identified in the enrolled patients.

Proband	Gene	Variant position (GRCh37/hg19) (GRCh38/hg38)	HGVS nomenclature	Exon/Total	Zygosity	MAF (gnomADv4) Grpmax FAF (95% confidence)	MAF (gnomADv4) in ME	ClinVar classification	In silico predictions SIFT, PP, MT, REVEL	ACMG classification (criteria)
1-II-2	*MYBPC3*	11:47364248-C-T 11:47342697-C-T	NM_000256.3:c.1505G>A; p.Arg502Gln	17/35	HET	0.00002549 (AF(A))	0	Pathogenic, likely pathogenic	T, D, D, U	Pathogenic (PM2_P, PM5, PS4, PP1_S)
2-II-1	*FLNC*	7:128482408-G-C 7:128842354-G-C	NM_001458.5:c.2245G>C; p.Val749Leu	14/48	HET	6.800e-7 (EU(NF))	0	VUS	D, T, D, U	VUS (PM2_P)
3-II-3	*ELAC2*	17:12917786-A-G 17:13014469-A-G	NM_018127.7:c.460 T>C; p.Phe154Leu	5/24	HOM	0	0.0001650	Pathogenic	D, D, D, U	Pathogenic (PM2_P, PS2, PS4)
4-II-1	*GAA*	17:78083846-AT-A 17:80110047-AT-A	NM_000152.5:c.1431delT; p.Ile477Metfs*43	9/20	ComHET	0	0	Pathogenic	NA	Pathogenic (PVS1, PM2_P, PM3_P, PP4)
		17:78086456-C-A 17:80112657-C-A	NM_000152.5:c.1834C>A; p.His612Asn	13/20	ComHET	0	0	VUS	D, D, D, U	VUS (PM2_P, PM5, PM3)
5-II-4	*TNNI3*	19:55667646-GC-G 19:55156278-GC-G	NM_000363.5:c.204delG; p.Arg69Alafs*8	5/8	HOM	0.00004297 (AdAm)	0	Pathogenic, likely pathogenic, VUS	NA	Likely pathogenic (PVS1, PM2_P)
6-II-1	*TNNI3*	19:55667646-GC-G 19:55156278-GC-G	NM_000363.5:c.204delG; p.Arg69Alafs*8	5/8	HOM	0.00004297 (AdAm)	0	Pathogenic, likely pathogenic, VUS	NA	Likely pathogenic (PVS1, PM2_P)
7-II-1	*MYBPC3*	11:47372811-C-T 11:47351260-C-T	NM_000256.3:c.271G>A; p.Asp91Asn	2/35	HET	0.000008390 (EU(NF))	0	VUS	D, D, D, B	VUS (PM2_P)
	*ADGRL2*	1:82302730-C-A 1:81837045-C-A	NM_001366006.2:c.61C>A; p.Pro21Thr	2/24	HOM	0.000007740 (EA)	0.0001678	VUS	U,NA,B,B	VUS (PM2_P)
	*NOL6*	9:33466597-C-G 9:33466599-C-G	NM_022917.5:c.2061G>C; p.Gln687His	16/26	HOM	0.0003241 (ME)	0.0008248	NA	D,D,D,U	VUS (PM2_P)
	*PIGQ*	16:626143-C-A 16:576143-C-A	NM_004204.5:c.831C>A; p.Asn277Lys	4/11	HOM	0	0	NA	U,NA,U,U	VUS (PM2_P)
8-II-2	*MYH7*	14:23898536-G-A 14:23429327-G-A	NM_000257.4:c.1159C>T; p.Leu387Phe	13/40	HET	0	0	Pathogenic	D, D, D, D	Likely pathogenic (PM2_P, PP1_M, PM1, PP3, PS4_P)
9-II-1	*FLNC*	7:128485076-C-T 7:128845022-C-T	NM_001458.5:c.3557C>T; p.Ala1186Val	21/48	HET	0	0	Pathogenic, likely pathogenic	D, D, D, U	Pathogenic (PM2_P, PS2, PS4)

*MAF* minor allele frequency, *gnomAD* the genome aggregation database, *ACMG* American College of Medical Genetics, *GUS* gene of uncertain significance, *SIFT* sorting intolerant from tolerant, *PP* PolyPhen-2, *MT* mutation taster, *HOM* homozygous, *HET* heterozygous, *ComHET* compound heterozygous, *VUS* variant of uncertain significance, *NA* not applicable, *T* tolerated, *D* deleterious, *U* uncertain, *B* benign, *ME* Middle Eastern, *AF(A)* African/African American, *EU(NF)* European (non-Finnish), *AdAm* Admixed American, *EA* East Asian.

### Two families (F01 and F02) manifesting sarcomere-related HCM

#### Family F01 with MYBPC3 as the plausible HCM-causing gene

**Figure 3:**
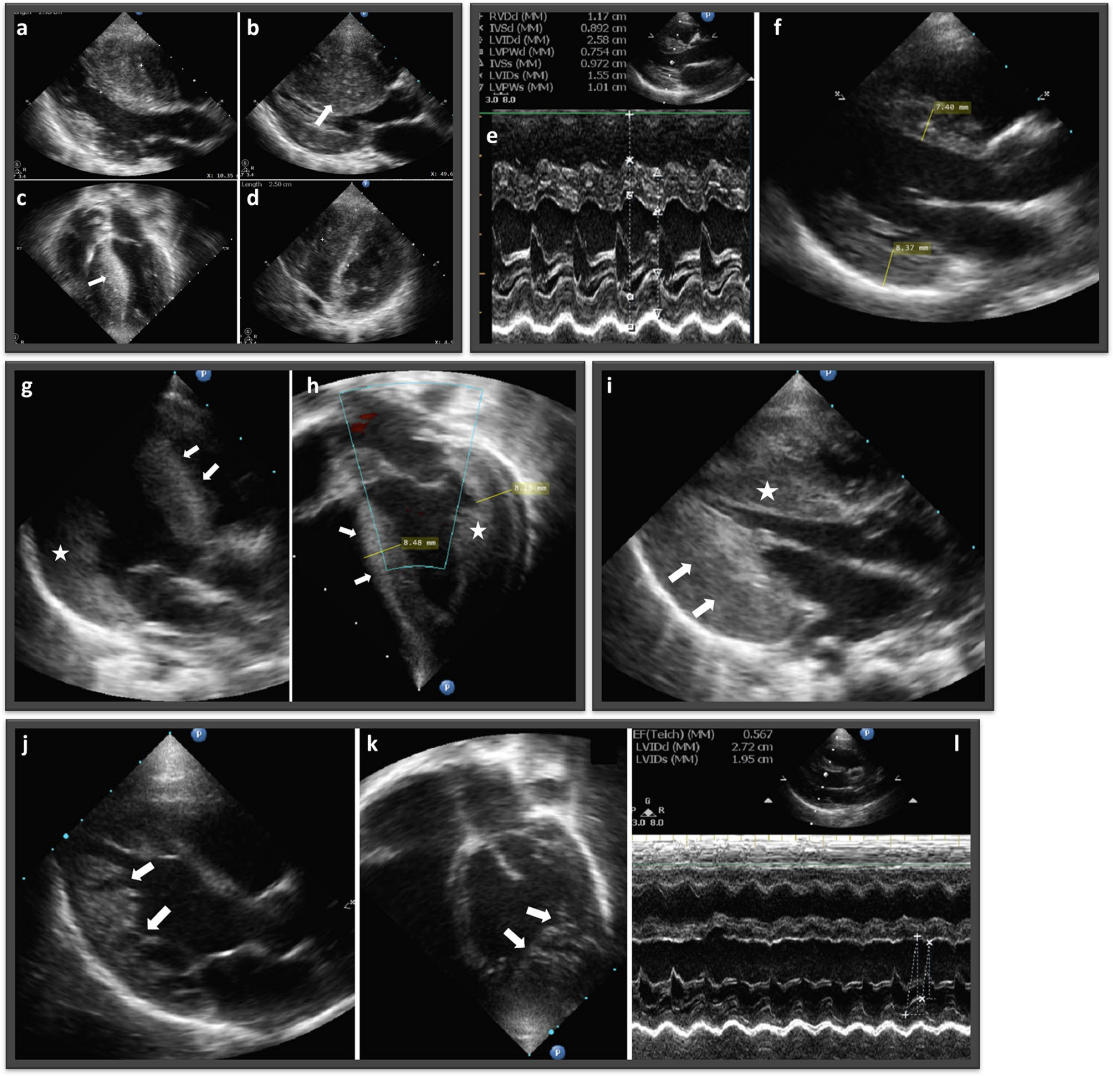
Echocardiographic images of patients with HCM and LVNC. (**a**–**d**) Patient in family F01. (**a**,**b**) Parasternal image in diastole and systole, respectively, showing the severely hypertrophic interventricular septum with diastolic dimension of 2.93 cm, and almost obliteration of the left ventricular lumen in systole. (**c**) is an image of the four-chamber view, and (**d**) of the short access view of the left ventricle showing the eccentric hypertrophy involving predominantly the interventricular septum. (**e**,**f**) Family 02 proband with hypertrophic cardiomyopathy. (**e**) M-mode echocardiography showed concentric hypertrophy with diastolic dimensions of the interventricular septum and posterior wall of 7.5–8.9 mm and preserved systolic function. (**f**) Two-dimensional echocardiographic image of the left ventricle showing hypertrophy of both septum (7.4 mm), and posterior wall (8.4 mm), with smooth, well-compacted myocardium. (**g**,**h**) Echocardiographic images of patient in family F03. (**g**) is a parasternal view, and (**h**) is an apical view of the left ventricle showing concentric left ventricular hypertrophy involving both the septum (white arrows) and the posterior wall (white star), the endocardial aspect is smooth, and with no evidence of left ventricular non-compaction. (**i**) Proband of Family F04. longitudinal echocardiographic image of the left ventricle in diastolic phase, showing severe hypertrophy of both interventricular septum (white star), and posterior wall of the left ventricle (white arrows), with well-compacted myocardium. (**j–l**) Proband in family F08. (**j**,**k**) Parasternal and apical views of the left ventricle showing non-compaction involving the apex and the posterior wall of the left ventricle (white arrows), and sparing the interventricular septum. (**l**) M-mode echocardiography showing mildly depressed left ventricular systolic function.

Proband 1-II-2 was a seven-year-old male with a history of acute lymphocytic leukemia. He was diagnosed with HCM during routine echocardiographic evaluation prior to initiation of chemotherapy for his leukemia, which showed interventricular septal hypertrophy with preserved systolic function (Table 1; Fig. 3a–d). Following successful treatment of his leukemia, the patient was on regular cardiac follow-up that showed a progressive increase in interventricular septum thickness, with new onset episodes of chest pain and dizziness. The patient was treated with beta-blockers and prophylactic implantation of an automatic defibrillator to prevent ventricular tachycardia and sudden death.

After ES analysis for the proband (1-II-2), a heterozygous missense variant (c.1505G>A;p.Arg502Gln) in the *MYBPC3* gene was labeled to be causing the patient’s HCM phenotype (Fig. 1a; Table 2; Fig. S2). This variant is rare in the population database gnomAD and the position of the original amino acid (*MYBPC3:*p.Arg502) is evolutionarily conserved across species. The ClinVar database has an entry for the variant (*MYBPC3:*p.Arg502Gln), reported by multiple independent submitters as either pathogenic or likely pathogenic. This missense variant has been previously documented in the literature among numerous patients with HCM, in both familial and sporadic forms^16,17^. Furthermore, *MYBPC3:*p.Arg502Gln was overrepresented in cases over controls^18^. Previously, at the same codon (p.Arg502), other missense variants (p.Arg502Trp, p.Arg502Leu, and p.Arg502Gly), have been reported in patients with HCM^19–21^. Collectively, we classified the variant *MYBPC3:*p.Arg502Gln as pathogenic (Table 2).

To investigate the status of this variant (*MYBPC3*:c.1505G>A) in other family members (1-I-1, 1-II-2, and 1-II-3; Fig. 1a), Sanger sequencing was performed. Accordingly, the sequence change (*MYBPC3*:c.1505G>A; Fig. S11a) was found to be absent from the proband’s reportedly unaffected sibling (1-II-3) and father (1-I-1), while the reportedly asymptomatic mother (1-II-2) was heterozygote. Consequently, evaluation of the mother (1-II-2) by echocardiography showed no signs of HCM.

#### Family F02 with HCM having FLNC as the plausible disease-causing gene

The proband in Family F02 (2-II-1) was referred to our institution at the age of 22 days due to respiratory difficulties and fatigue during feeding. Upon evaluation, the infant was found to have concentric HCM with dimensions of 6.5 mm and 7 mm in the interventricular septum and posterior wall, respectively (Table 1; Fig. 3e,f). The systolic function was normal with an ejection fraction of 70%. Conservative management was implemented, which included increased caloric intake and regular follow-up. At 16 months of age, the patient's cardiac condition was stable without disease progression.

The trio-based ES revealed a maternally inherited heterozygous (c.2245G>C;p.Val749Leu) substitution in *FLNC* in the proband (2-II-1) (Fig. 1b; Table 2). The *FLNC*:c.2245G>C is not a common change in the screened populations in gnomADv4. Despite the fact that *FLNC*:c.2245G>C has not been reported in the literature before, it has a record in ClinVar by one submitter as a VUS. Taken together, the current knowledge about *FLNC*:c.2245G>C is insufficient to conclude its pathogenicity. Altogether, we classify this variant as VUS. Unfortunately, the reportedly asymptomatic mother, a carrier of the *FLNC*:c.2245G>C, was unavailable for cardiovascular disease evaluation (Table 2). No other biallelic variants were found in proband (2-II-1) to be implicated in causing CMP (Table S4).

### Family F03 and F04 exhibiting phenocopies of HCM

#### Family F03 showing HCM with a pathogenic variant in ELAC2 gene

The proband from family F03 (3-II-3), a 6-month-old male infant, was brought to our attention due to heart failure symptoms that included respiratory distress, decreased feeding, and failure to gain weight. His medical history revealed that he had an older male sibling who died of heart failure in infancy. An echocardiogram revealed concentric HCM and mild systolic function depression, with an ejection fraction of 54% (Table 1; Fig. 3g,h). The interventricular septum and posterior wall were both hypertrophic, with measurements of 8.5 and 8.2 mm, respectively. The child unfortunately passed away a month after the diagnosis due to decompensated heart failure following a viral infection.

ES analysis on the proband, his parents, and his healthy sibling revealed that the proband was homozygous for c.460T>C variant in *ELAC2* (Fig. 1c; Table 2; Fig. S3). The *ELAC2:*c.460T>C was only detected in one out of 1,613,834 alleles in the gnomADv4 database. The variant *ELAC2:*p.Phe154Leu has been reported in the ClinVar and literature in a number of patients with combined oxidative phosphorylation defect type 17^22,23^. *ELAC2:*c.460T>C co-segregated with the disease phenotype among affected siblings from several families^24^. Prior in vitro functional analysis of fibroblasts from patients harboring *ELAC2*:p.Phe154Leu variant has shown compromised ELAC2 activity^22,25^. Unfortunately, due to the proband's death, we were unable to test for mitochondrial oxidative phosphorylation function. Collectively, we classify *ELAC2*:p.Phe154Leu as pathogenic (Table 2).

#### Family F04 with GAA labeled as the causative gene

Proband F4-II-1 was a 2-month-old male infant diagnosed with bilateral clubfoot, who presented with symptoms of feeding difficulties, tachypnea, and dyspnea since birth. He was admitted to the neonatal intensive care unit for 4 weeks, where an echocardiogram showed severe concentric left ventricular hypertrophy with normal systolic function (Table 1; Fig. 3i). Unfortunately, despite medical intervention, the infant died of respiratory failure at the age of 4 months, following a respiratory infection.

After conducting trio-based ES to determine the genetic cause of the proband’s (4-II-1) clinical picture, biallelic variants in *GAA* were revealed (Fig. 1d; Table 2; Fig. S4). The paternally inherited variant, *GAA*:c.1431delT, deletes thymine at position c.1431 in exon 9 of the NM_000152.5 transcript. This deletion will lead to an out-of-frame shift in the translated protein. This shift starts with substituting isoleucine at codon 477 with methionine, followed by subsequent substitutions, and ends up with a premature stop codon after 43 amino acids downstream. The truncated *GAA* is expected to undergo NMD. The p.Ile477Metfs*43 variant has not been reported in population databases. Multiple pathogenic loss-of-function (LoF) variants have been reported downstream of p.Ile477Metfs*43. Homozygous *GAA*:p.Ile477Metfs*43 has been reported in patients with type II glycogen storage disease (Pompe disease)^26,27^. Taken together, this variant meets the criteria for a pathogenic classification (Table 2).

On the other hand, the maternally inherited missense variant, *GAA*:c.1834C>A;p.His612Asn, has neither been reported in the literature nor the gnomADv4 database (Fig. 1d; Table 2). Collectively, the current body of evidence is insufficient to deduce the pathogenicity of *GAA*:c.1834C>A. Subsequently, *GAA*:p.His612Asn is classified as a VUS, in alignment with the variant’s ClinVar entry (Table 2).

The discovery of a pathogenic variant in *GAA* implicated Pompe disease as the likely cause of the proband 4-II-1’s clinical picture. Regrettably, the diagnosis of Pompe disease was not further confirmed due to the patient’s demise prior to the release of the genetic results.

### Three families (F05, F06 and F07) exhibiting DCM

#### Families F05 and F06 with DCM harbor the same lethal likely pathogenic variant in TNNI3

**Figure 4:**
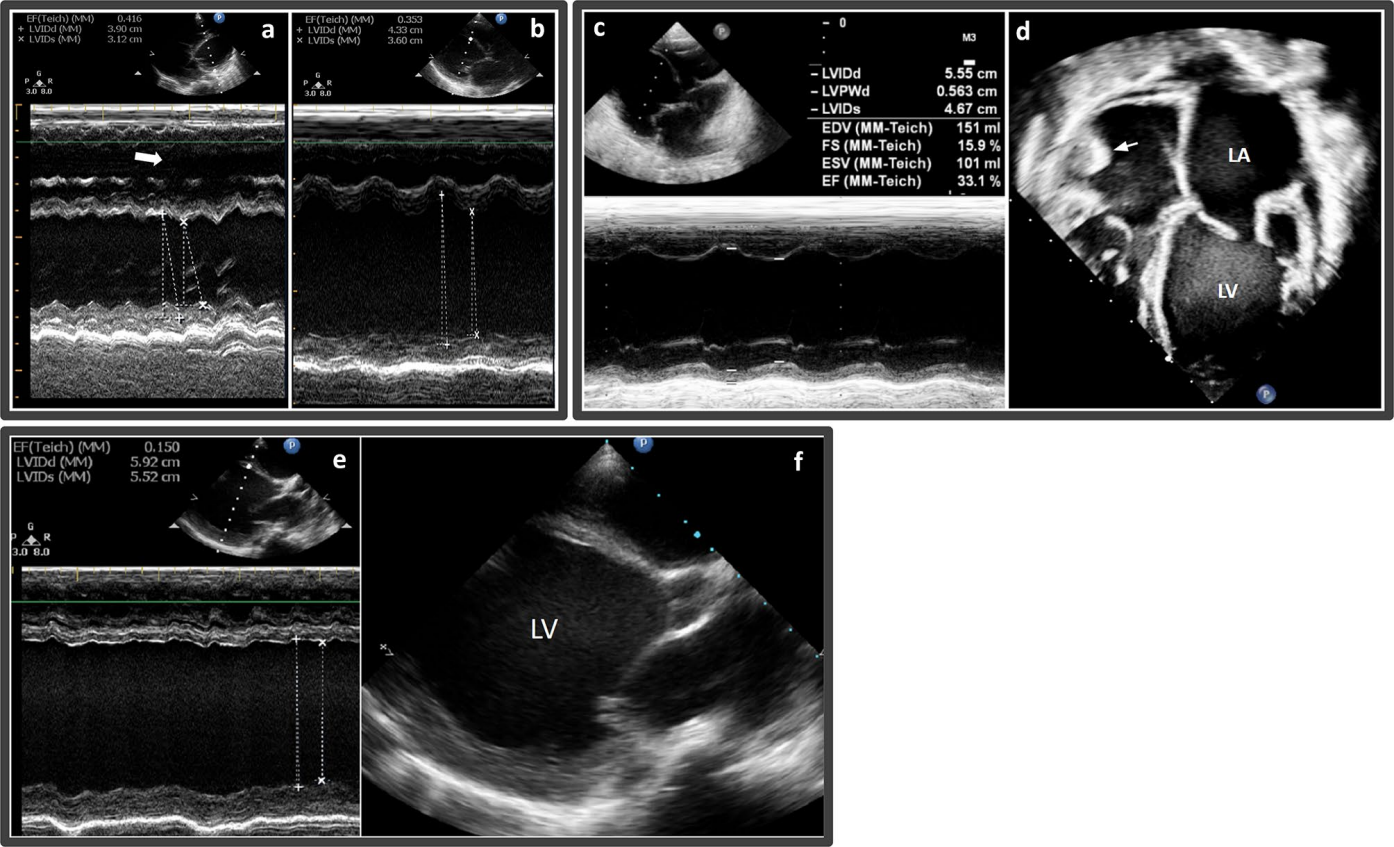
Echocardiographic images of patients with DCM. (**a**,**b**) M-mode echocardiographic images for 5-II-4 and 5-II-5 in Family F05. (**a**) 5-II-4, severe left ventricle dilatation with systolic dysfunction, with ejection fraction of 41%. The right ventricle is also dilated (white arrow) due to the presence of secundum ASD. (**b**) 5-II-5, severe left ventricle dilatation with systolic dysfunction, with an ejection fraction of 35%. **c-d** M-mode (**c**) and apical four-chamber (**d**) echocardiographic image of proband F06, showing severely dilated left ventricle (LV) and left atrium (LA), and depressed ventricular systolic function with ejection fraction (EF) of 33.1%. A right atrial thrombus is also seen (arrow). (**e**,**f**) Proband of Family F07. (**e**) M-mode and (**f**) longitudinal echocardiographic image of the left ventricle (LV) of a 2-year-old girl with dilated cardiomyopathy, showing severely dilated left ventricle, and severely depressed ventricular systolic function with ejection fraction (EF) of 15%.

Two families (F05 and F06; Fig. 1e,f) had similar presentations of DCM in their children (Fig. 4a–d). Prior to the cardiac evaluation, all affected individuals exhibited comparable symptoms, such as breathing difficulties, feeding problems, and rapid breathing. Later cardiac evaluations in both families revealed dilated left ventricles and reduced systolic function (Table 1; Fig. 4a–d). Family F05 had four children who were diagnosed with DCM between the ages of 6–9 months and did not survive beyond 18 months. The proband in family F06 was diagnosed at the age of 10 months and died at 13 months despite receiving anti-failure medication. Notably, neither family had a history of cardiac disease in previous generations. Noteworthy, F05 and F06 were not related based on the family records.

To investigate the genetic basis of their DCM presentation, we performed ES and trio-based ES for the probands 5-II-4 and 6-II-1 with their parents, respectively (Fig. 1e,f). Remarkably, we identified a homozygous truncating variant c.204delG (p.Arg69Alafs*8) in *TNNI3* in both probands of unrelated families (Table 2). This variant resulted in the deletion of one nucleotide at c.204 in exon five of the transcript NM_000363.5. Thus, the reading frame was disrupted; starting with a substitution of p.Arg69 for Ala and a subsequent early stop codon after eight amino acids. Since this variant is located in exon five out of ten, a nonsense-mediated mRNA decay was predicted to occur. The variant’s allele count is rare in the population database gnomADv4 without any reported homozygotes. This indicates that the *TNNI3*:p.Arg69Alafs*8 variant is not a common benign variant in the homozygous state in the screened populations (Table 2).

Since the parents of both probands (5-II-4 and 6-II-1) were unaffected and heterozygotes for the variant (*TNNI3*:c.204delG), an autosomal recessive (AR)-co-segregation of this variant was anticipated to be disease-causing. Sanger sequencing of this variant was conducted on the available family members from F05 (Table 2; Fig. 1e; Figs. S5, S6). Co-segregation analysis revealed that the proband’s (5-II-4) affected (5-II-5) and unaffected (5-II-3) siblings were homozygous and heterozygous for this sequence change (*TNNI3*:c.204delG), respectively (Fig. S11b). Thus, our study demonstrated that this variant's inheritance pattern follows an AR mode-of-inheritance.

This homozygous variant (*TNNI3*:p.Arg69Alafs*8) was previously reported in the literature in patients with DCM^28–30^.Furthermore, a prior functional study documented the absence of *TNNI3* at the mRNA and protein levels in the cardiac biopsies of a patient with the p.Arg69AlafsTer8 variant in the homozygous state, suggesting a possible degradation of TNNI3 by NMD^30^. This variant is listed in the disease database ClinVar (Variation ID: 179447, last accessed 2024-05-30), but with conflicting interpretations of variant of uncertain significance to pathogenic among reputable sources. Our analysis provides further evidence supporting the pathogenic implication of this variant and collectively, we classified this variant as likely pathogenic (Table 2).

#### Family F07 had DCM with inconclusive genetic findings

The proband from family F07 (7-II-1) was diagnosed with autistic spectrum disorder and mild developmental delay at the age of two years and had decreased exercise tolerance. He was found to have DCM with systolic dysfunction on echocardiography (Table 1; Fig. 4e,f) and, despite being on anti-failure medications, the patient died at the age of 3.5 years due to decompensated heart failure.

We performed trio-based ES analysis to decipher the genetic factors underlying proband’s 7-II-1 clinical manifestations (Fig. 2a; Table S9). We identified four candidate VUSs, namely *MYBPC3*:p.Asp91Asn, *ADGRL2*:p.Pro21Thr; *NOL6*:p.Gln687His , and *PIGQ*:p.Asn277Lys (Table 2; Figs. S7, S8; Supplementary Text S1; Table S9). However, none of these genes align completely with the clinical manifestations of the proband 7-II-1.

### LVNC in family F08 revealed *MYH7* as a possible implicated gene

Due to a family history of sibling death during the neonatal period with suspicion of cardiomyopathy, proband (8-III-2) was evaluated soon after birth. Although the infant showed no signs of heart disease, echocardiography revealed the presence of LVNC in the apex and posterior wall of the left ventricle, with sparing of the interventricular septum (Table 1; Fig. 3j–l). The maximum systolic non-compaction to compaction thickness (NC/C) was 2.8, measured at the apical aspect of the posterior wall of the left ventricle, and the systolic function was mildly depressed with an ejection fraction of 56%. Regular outpatient follow-ups were conducted with the most recent follow-up at the age of 2 years, with a stable condition reported. Later, a third child (8-III-3) was born into the family and was also found to have LVNC with more significant systolic dysfunction. Unfortunately, the patient 8-III-3 died at the age of 6 months due to decompensated heart failure.

ES analysis was performed for proband (8-III-2), his parents (8-II-1 and 8-II-2), and his younger brother (8-III-3) (Fig. 2b). The analysis identified a shared paternally inherited heterozygous variant-of-interest (*MYH7*: p.Leu387Phe) in both siblings (8-III-2, and 8-III-3; Fig. S9). The variant (MYH7:p.Leu387Phe) is located in a hotspot region for LVNC which encompasses the adenosine triphosphatase (ATP) region of the head domain in the β-myosin heavy chain (*MYH7*)^31^ (Table 2). This domain has been identified as a functional/hotspot domain in MYH7^32^. The *MYH7*:p.Leu387Phe has not been reported in any population database and it has a pathogenic classification in ClinVar by one submitter in the context of HCM. Cases with LVNC harboring *MYH7*:p.Leu387Phe have been previously documented^33^. Taken together, the *MYH7*:p.Leu387Phe variant is classified as likely pathogenic (Table 2).

Accordingly, the paternally inherited variant prompted us to do an echocardiographic evaluation for the father which revealed him to have LVNC with mildly depressed systolic function with an ejection fraction of 48%. Thereby, further investigation of the family F08’s medical history disclosed a positive history of cardiac defects on the paternal aunt’s side. Two daughters of the paternal aunt presented with heart disease after birth (8-III-6 and 8-III-7; Fig. 2b; Fig. S9). One daughter (8-III-6) had mild LVNC with mild right ventricular hypoplasia requiring medical therapy, while the other (8-III-7) had biventricular non-compaction and severe right ventricular hypoplasia requiring early intervention with ductus arteriosus stenting, with significant improvement of right ventricular size with time. However, both sisters (8-III-6 and 8-III-7) had preserved left ventricular systolic function and had no cardiac symptoms at the last follow-up at the ages of 5 and 2 years, respectively.

Samples were collected for genetic testing from the paternal aunt (8-II-4), an affected female cousin (8-III-7), and an unaffected male cousin (8-III-4). The testing revealed that all three individuals were carriers for the *MYH7*:p.Leu387Phe variant (Fig. 1b; Fig. S9; S11c). Following up on the discovery of a likely pathogenic variant in *MYH7* in the reportedly asymptomatic individuals, cardiac evaluation was carried out. Consequently, the reportedly asymptomatic aunt (8-II-4) turned out to have mild LVNC, while the unaffected cousin (8-III-4) did not have any cardiac abnormalities.

### RCM in family F09 with *FLNC* labeled as the implicated gene

**Figure 5:**
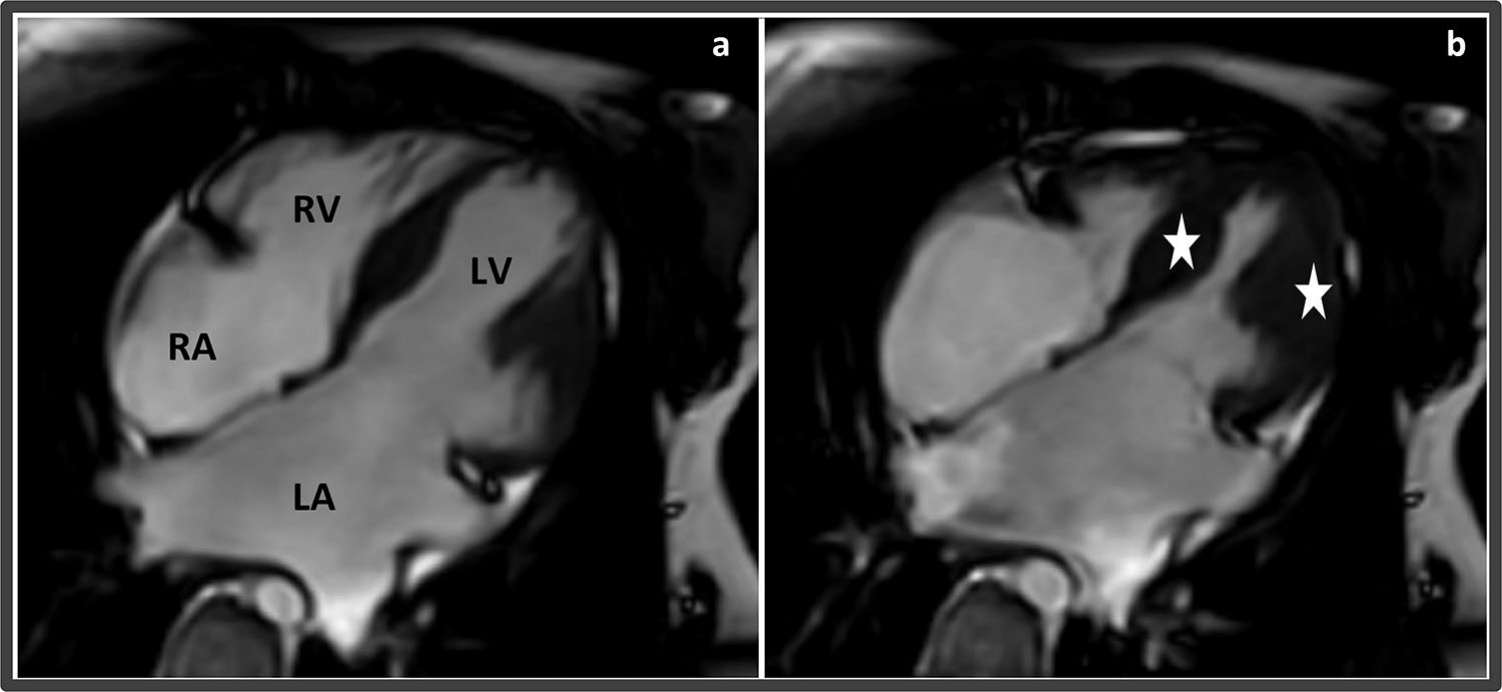
Cardiac magnetic resonance image of proband in F09 with RCM. (**a**) Cardiac magnetic resonance image of 4-chamber view in diastolic, and (**b**) systolic phases, showing Bi-atrial dilatation, with preserved systolic function. Mild thickening of the mid-section of interventricular septum and left ventricular free wall is also noted (white stars). *LA* left atrium, *LV* left ventricle, *RA* right atrium, *RV* right ventricle.

In Family F09, proband 9-II-1 was a 10-year-old girl who presented with progressive dyspnea, exercise intolerance, and palpitations since the age of 8 years. An echocardiographic evaluation showed bi-atrial enlargement and normal ventricular sizes and systolic function indicating diastolic dysfunction, consistent with RCM. This was confirmed by a cardiac magnetic resonance imaging study, which revealed bi-atrial enlargement, dilated caval veins, normal ventricular muscle mass, and systolic function, with no pericardial thickening (Fig. 5).

To elucidate the molecular etiology of the proband’s (9-II-1) RCM presentation, trio-based ES was conducted and a de novo (c.3557C>T;p.Ala1186Val) variant in *FLNC* was revealed (Fig. 2c; Table 2). The *FLNC*:c.3557C>T sequence change is not found in gnomADv4 database, reflecting that this is not a frequent benign variant in the general population. A consensus pathogenic/likely pathogenic classification of the *FLNC*:p.Ala1186Val has been submitted to ClinVar database by nine institutions. The p.Ala1186Val variant has been reported in the literature in patients with isolated myofibrillar myopathy, and RCM accompanied by congenital myopathy^34–36^. Collectively, we classified *FLNC*:p.Ala1186Val as pathogenic (Table 2).

## Discussion

In this molecular investigation of pediatric CMP in Jordan, we incorporated ES analysis for 14 patients from nine families with different subtypes of the condition. Our investigation revealed pathogenic and likely pathogenic variants in 77.8% of families (7 out of 9), with clustering in sarcomere-related genes. Nonetheless, phenocopies of sarcomeric HCM were also detected. The remaining 22.2% of the families (2 out of 9) had VUSs.

HCM was the most prevalent subtype of cardiomyopathies in our cohort, identified in four families (F01, F02, F03, and F04). Nevertheless, only two families with HCM (F01 and F02) had candidate variants in sarcomere-related genes, namely *MYBPC3* and *FLNC* (Fig. S1). Family F02 harbored a VUS in *FLNC* (p.Val749Leu). Pathogenic variants in *FLNC* have been linked not only to causing myopathies but also to isolated HCM and RCM, as described in families F02, and F09, respectively^36,37^.

The second sarcomere-related HCM was associated with *MYBPC3.* Pathogenic variants in *MYBPC3* were accounted for as one of the leading causes of HCM in previous studies^12,38^. In family F01, we detected a pathogenic variant (p.Arg502Gln) in *MYBPC3*. Although the variant (*MYBPC3*:p.Arg502Gln) in proband 1-II-2 was maternally inherited, the mother was then asymptomatic. This observation could be indicative of incomplete, gender-related, or age-related penetrance. Several studies, comparing the HCM penetrance relative to age of diagnosis and gender among individuals with pathogenic variants in *MYBPC3*, found that females tend to exhibit delayed HCM onset^39–41^. Accordingly, the mother (1-I-2) should undergo regular surveillance for any future signs of HCM.

Phenocopies of sarcomeric HCM were also observed in two families (F03 and F04). Proband 3-II-3 suffered from infantile-onset HCM and carried a pathogenic variant (p.Phe154Leu) in *ELAC2.* Previously, the patients harboring this variant experienced lactic acidosis, mitochondrial complex I deficiency, and, similarly, an infantile-onset HCM^23,24^. A prior study found that homozygous *ELAC2*:p.Phe154Leu was associated with a poor prognosis, leading to infantile CMP-related death, as observed in proband 3-II-3 and his older brother^24^. Autozygosity mapping of 16 unrelated Arab families harboring *ELAC2*:p.Phe154Leu pinpointed a loss-of-heterozygosity in the flanking region. Therefore, *ELAC2*:p.Phe154Leu may be a founder variant in the Arab population^24^. Such findings can assist in the future design of region-specific targeted variant panels. In societies with limited resources, where next-generation sequencing (NGS) remains a burden, testing for targeted variants, at least as a first-tier approach, offers a more affordable solution due to its lower cost.

The other HCM phenocopy was discovered in F04. At the time of recruitment, proband (4-II-1) presented with HCM and was found to harbor biallelic variants (p.His612Asn, and p.Ile477Metfs*43) in *GAA.* The cardinal features of GAA-related disorder include muscle weakness, hypotonia, and infantile-onset HCM that mimic sarcomeric HCM^42^. Several Arab patients with *GAA*:p.Ile477Metfs*43 variant were reported to have an infantile-onset Pompe disease^26,27,43^. For instance, an Egyptian patient with the same homozygous variant (*GAA*:p.Ile477Metfs*43) was diagnosed with Pompe disease, featuring reduced GAA activity, cardiomyopathy, and hypotonia^43^. Although the diagnosis of Proband (4-II-1) with Pompe disease was not confirmed by laboratory testing, it is appropriate to make the diagnosis based on genetic results and clinical features of HCM, feeding difficulty and dyspnea that can be attributed to muscle weakness and hypotonia. Notably, the early and precise identification of HCM phenocopies can have a positive impact on the course of the disease by incorporating specific treatment regimens, such as enzyme replacement therapy and immunosuppressive treatment in patients with Pompe disease^10^. This finding emphasizes the need to screen for HCM phenocopies as part of the clinical management of patients with severe HCM. These findings pinpoint the importance of expanding the HCM genetic testing panels to also encompass HCM-related phenocopies. This is of relevance as there has been a discrepancy among various molecular diagnostic institutions regarding the inclusion of phenocopy genes within their HCM and CMP panels. This discrepancy underscores the importance of expanding HCM genetic testing panels to incorporate genes associated with HCM-related phenocopies.

Three recruited families had DCM (F05, F06, and F07). Among these, two unrelated families (F05 and F06) shared the same homozygous likely pathogenic variant (*TNNI3:*p.Arg69Alafs*8). Intriguingly, the role of homozygous LoF variants in the context of *TNNI3*-triggered CMP has been associated with early-onset AR-DCM^44^. In F05 and F06, the co-segregation analysis of the variant (*TNNI3*:p.Arg69Alafs*8) was also indicative of AR-inheritance. This homozygous variant (*TNNI3:*p.Arg69Alafs*8) has been reported in several families with AR-DCM^28–30^. These previously described cases, along with our study subjects, had comparable clinical scenarios in terms of manifesting early-onset and severe presentations of DCM^28^.

Generally, the least prevalent subtypes of pediatric cardiomyopathies are LVNC and RCM, each comprising 3% of cases^45^. Here, one family (F08) exhibited a familial occurrence of LVNC, presenting in seven family members across two generations (Fig. 2). The affected individuals in family F08 manifested with variable severity and a broad range of ages at diagnosis, spanning from infancy to adulthood. The same patterns were documented in LVNC and, in particular, MYH7-triggered LVNC^46^. The likely pathogenic variant in F08 (*MYH7*:p.Leu387Phe) was previously reported in a state of triple-heterozygous inheritance in siblings afflicted with LVNC, implying a plausible oligogenic inheritance pattern^33^. This presentation of LVNC was similarly recapitulated in a mouse model harboring the same triple-heterozygous variants^33^. Conversely, our ES analysis did not uncover any other common variants that can predispose the affected members in F08 to an oligogenic-driven LVNC phenotype. Noteworthy, after conducting a cascade of genetic and clinical testing on F08, we helped in diagnosing the then-reportedly asymptomatic paternal aunt (8-II-4) with LVNC and discovering the variant in her contemporary asymptomatic son (8-III-4). Whether the individual (8-III-4) will manifest LVNC later in life or remain asymptomatic is uncertain since both incomplete and age-related penetrance have been described in *MYH7*-related disorders^47^. Moving forward, both individuals (8-II-4 and 8-III-4) are advised to undergo regular cardiac follow-ups to mitigate any potential LVNC-associated complications. Further, this highlights the need for implementing genetic testing for at-risk individuals in families with a history of cardiomyopathies, allowing for proactive clinical monitoring for carriers and early interventions.

As for the RCM, family F09 had an only child (9-II-1) with a de novo pathogenic variant (p.Ala1186Val) in *FLNC,* diagnosed at the age of 8 years. This variant (p.Ala1186Val) has been described in patients with early-onset RCM accompanied by congenital myopathy and arthrogryposis^35^ as well as isolated RCM^36^. In our investigation, another proband with HCM (2-II-1) had a maternally inherited VUS (p.Val749Leu) in *FLNC*. Reduced penetrance of variants in *FLNC* has been shown in families with mild HCM, which may explain why the mother of proband 2-II-1 is currently asymptomatic^48^. The prevalence of FLNC-related cardiomyopathies was estimated to be 8% for RCM and 1.3% for HCM^37^. While *FLNC*-associated cardiomyopathies have been described as concomitant with myopathies, the probands 2-II-1, and 9-II-1 have not (yet) manifested any other co-morbidities beyond their cardiac phenotype. These findings show that *FLNC* is pleiotropic and implicated in a wide range of cardiac and muscular disorders with variable expressivity, age of onset, and penetrance. Therefore, close monitoring of these individuals is necessary to detect any future complications.

While ES analysis was able to identify the molecular underpinnings in most of the cases, inconclusive findings were observed in two families (F02 and F07). A recessive mode of inheritance was anticipated for F02, given the early onset of CMP and that the parents of proband 2-II-1 were then asymptomatic and consanguineous. Regrettably, we were unable to find any bi-allelic, particularly homozygous, variants that could be causal for cardiomyopathies in F02 (Table S4). The identified VUS (p.Val749Leu) in *FLNC* currently lacks conclusive evidence to be labeled as the primary genetic cause for the HCM presentation in proband 2-II-1. Future research is needed to conclude the pathogenicity of this variant or to implicate yet-to-be-discovered genes that may cause CMP.

In family F07, proband (7-II-1) exhibited a complex clinical profile, suffering from DCM, autism spectrum disorder, and developmental delay. After ES analysis, four candidate VUSs were identified (Table 2; Supplementary Text S1; Table S9). These variants may partially explain some aspects of the proband’s manifestations, albeit deviating from the typical symptoms associated with the described genes (Supplementary Text S1; Table S9). While ES analysis can give valuable insights into the genetic etiology of cardiomyopathies, it has inherent detection limitations. Thus, further rigorous research is required to discover variants uncovered by ES and to reinterpret the detected VUSs and genes of uncertain significance.

In conclusion, this study sheds light, for the first time, on the genetic landscape of cardiomyopathies in Jordan. Interestingly, we highlighted the clinical utility of ES analysis in identifying (likely) pathogenic variants in 77.8% of the families with CMP, mostly clustering in sarcomere-related genes. Moreover, we emphasized the importance of utilizing comprehensive genetic testing for patients with CMP to reveal potential CMP-related phenocopies. We also prompted a cascade of genetic testing in at-risk relatives and helped detect the candidate variants in some asymptomatic individuals. These findings can significantly impact the management of such cases, introduce early interventions, and improve their prognosis. Finally, our work provides a starting point for elucidating the molecular etiologies of cardiomyopathies in Jordan. Our findings can contribute to the future development of region-specific targeted variant panels. In communities facing resource constraints, where NGS poses financial challenges, targeted variant testing emerges as a cost-effective option, particularly as a primary screening method.

## Methods

### Study subjects

Nine families with unaffected and affected family members with CMP were recruited into this study. Patients with pediatric CMP were recruited from the pediatric cardiology clinic during the period of 2019–2022. Diagnosis of CMP was done by detailed clinical history, and physical examination and confirmed by echocardiography (Philips Hd 11 Xe ultrasoundsystem; Philips Healthcare). This study adhered to the tenets of the Declaration of Helsinki and was approved by the institutional review Board (IRB) of Jordan University Hospital (Protocol code 2018/198, 26/6/2018). Prior to the subject enrolment, an informed consent form was secured from each recruited individual or their legal guardian.

### Sample collection, and genetic testing

**Figure 6:**
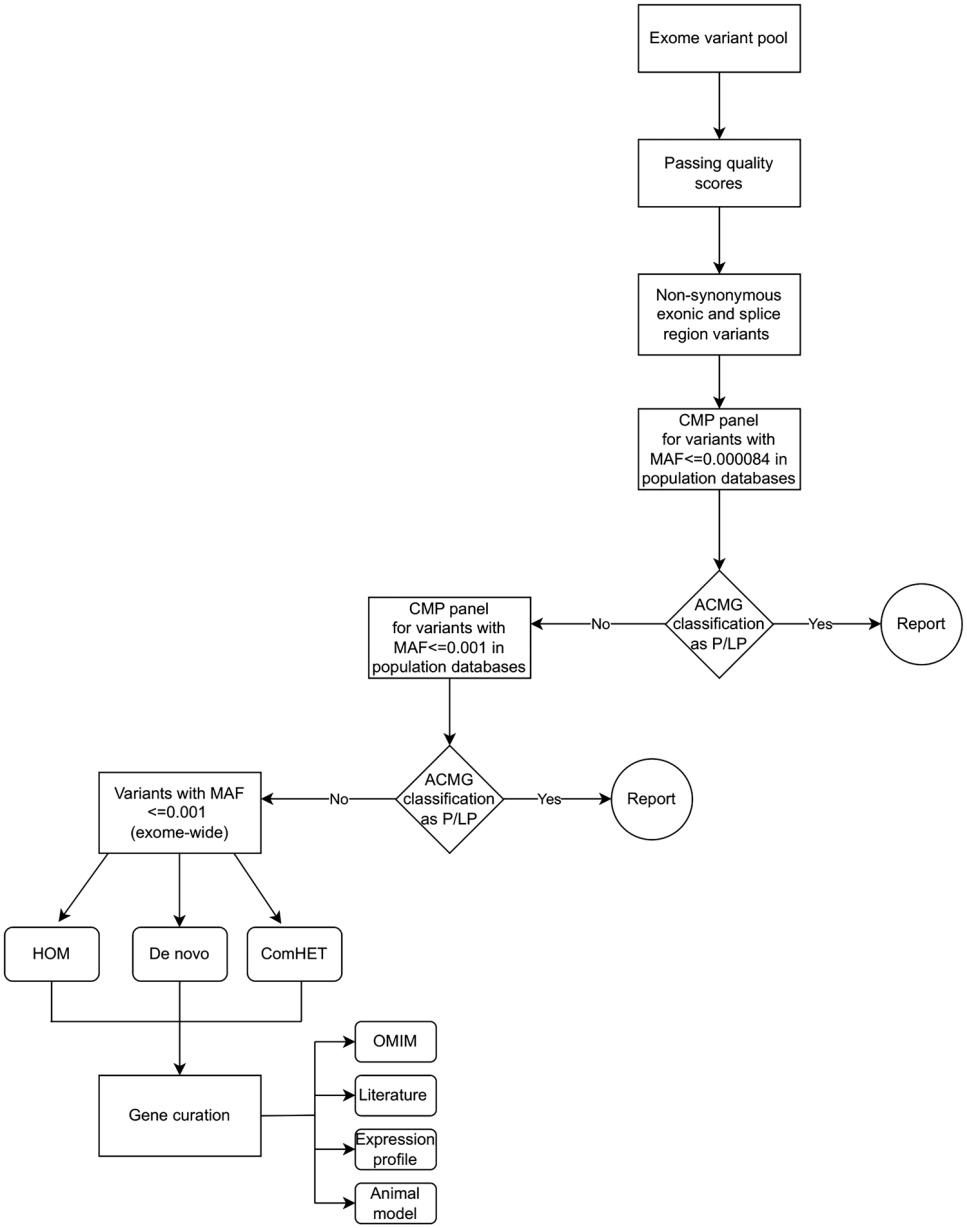
Flowchart illustrating the multi-step filtration approach to filter and select the described variants. *CMP* cardiomyopathy, *MAF* minor allele frequency, *HOM* homozygous, *ComHET* compound heterozygous, *ACMG* American College of Medical Genetics guidelines, *P/LP* pathogenic/likely pathogenic.

Venous blood samples were collected in 5 mL EDTA tubes from each recruited individual (Figs. 1, 2). DNA was extracted from blood samples using the Wizard purification Kit in compliance with Promega’s recommended protocol (Madison, Wisconsin, United States). ES for families (F02-F04, and F06-F09) was conducted as described by Azab et al.^49^, while ES for families (F01 and F05) was performed as highlighted by Tawalbeh et al.^50^ (Fig. 1). The ES quality metrics are available in Tables S1 and S2. Our filtration was confined to the rare non-synonymous exonic and splice‑site variants that exhibited high-quality sequence reads, passing GATK Variant Score Quality Recalibration (VSQR), with a genotype quality score (GQ) ≥ 30, a minimum of 20 total reads, and an alternate allele ratio of ≥ 30% (Fig. 6). We adopted a multi-step filtration approach to account for mono-and-bi-allelic modes of inheritance in cardiomyopathies. Our first-tiered approach was built to screen for (likely) pathogenic variants in CMP-related genes (Table S12). As the majority of the CMP cases are inherited in a dominant fashion, the filtration process began by running the CMP panel for variants with a minor allele frequency (MAF) of ≤ 0.000084 in population databases, accounting for the dominant mode of inheritance, based on the highest recommended filtration threshold for CMPs according to Whiffin et al.^51^. If no variants were classified as pathogenic or likely pathogenic according to the ACMG guidelines, the MAF threshold was increased to ≤ 0.001 in the CMP panel^15^. The MAF threshold of ≤ 0.001 has been previously adopted by other regional studies studying CMPs in under-represented consanguineous populations^13,25,44^. This is to account for potential recessive inheritance and to allow for the detection of potential private/founder variants in highly consanguineous populations such as Jordan. As a second-tiered approach when no (likely) pathogenic variants were identified in the prior steps, further screening was conducted for homozygous (HOM), compound heterozygous (ComHET), and de novo variants with a MAF of ≤ 0.001. Finally, the genes harboring these variants were curated against literature, expression profiles, the OMIM database, and gene function to assess their relevance and potential role in the disease (Fig. 6; Tables S3–S11). The filtered de novo variants were called if the proband and the parents had ≥ 30% and < 3% of the alternate allele ratios, respectively. Moreover, we recorded compound heterozygous variants if each biallelic variant was inherited from a different parent. All recorded variants were then verified using Integrative Genomics Viewer (IGV). The IGV pileups of the candidate variants are shown in Figs. S2–S10. Selected genes with candidate variants (Table S13) underwent co-segregation analysis by Sanger sequencing for individuals highlighted in Figs. 1 and 2 and Fig. S11.

### Supplementary Information

Supplementary Figures.

Supplementary Tables.

Supplementary Information 3.

## Data Availability

Any relevant data supporting the findings of this work are available within the paper and the provided supplementary material. Upon a reasonable request, any further required data supporting the findings of this work can be provided by the corresponding author.
