# Supplementary material for: Unraveling the genetic tapestry of pediatric sarcomeric cardiomyopathies and masquerading phenocopies in Jordan
Source: Sci Rep. 2024 Jul 2;14:15141. doi: 10.1038/s41598-024-64921-9 (PMC11219879; doi:10.1038/s41598-024-64921-9)
Supplement: Supplementary file 1 — Supplementary Figures. [file 41598_2024_64921_MOESM1_ESM.pdf]

Unraveling the genetic tapestry of pediatric sarcomeric cardiomyopathies and masquerading phenocopies in Jordan

Bilal Azab[1,2,†,*], Dunia Aburizeg[2,†], Sherin T. Shaaban[3], Weizhen Ji[4], Lina Mustafa[2], Nooredeen Jamal Isbeih[2], Amal Saleh Al-Akily[2], Hashim Mohammad[2,] Lauren Jeffries[4], Mustafa Khokha[4,5], Saquib Lakhani[4], Iyad Al-Ammouri[6,**].

1-Division of Pathology and Laboratory Medicine, Phoenix Children's Hospital, Phoenix, Arizona 85016, USA

2- Department of Pathology and Microbiology and Forensic Medicine, School of Medicine, The University of Jordan, Amman 11942, Jordan

3-Department of Biology and Biotechnology, Faculty of Science, American University of Madaba, Madaba, 11821, Jordan

4- Yale University School of Medicine, Department of Pediatrics, Pediatric Genomics Discovery Program, New Haven, CT, 06510, USA

5- Yale University School of Medicine, Department of Genetics, New Haven, CT, 06510, USA

6-Department of Pediatrics, School of Medicine, The University of Jordan, Amman 11942, Jordan

[†] Bilal Azab and Dunia Aburizeg contributed equally to this work

[*]Corresponding author: Bilal Azab, Division of Pathology and Laboratory Medicine, Phoenix Children's Hospital, Phoenix, Arizona 85016, USA, bazab@phoenixchildrens.com

[**]Co-corresponding author: Iyad Al-Ammouri, Department of Pediatrics, School of Medicine, The University of Jordan, Amman 11942, Jordan,

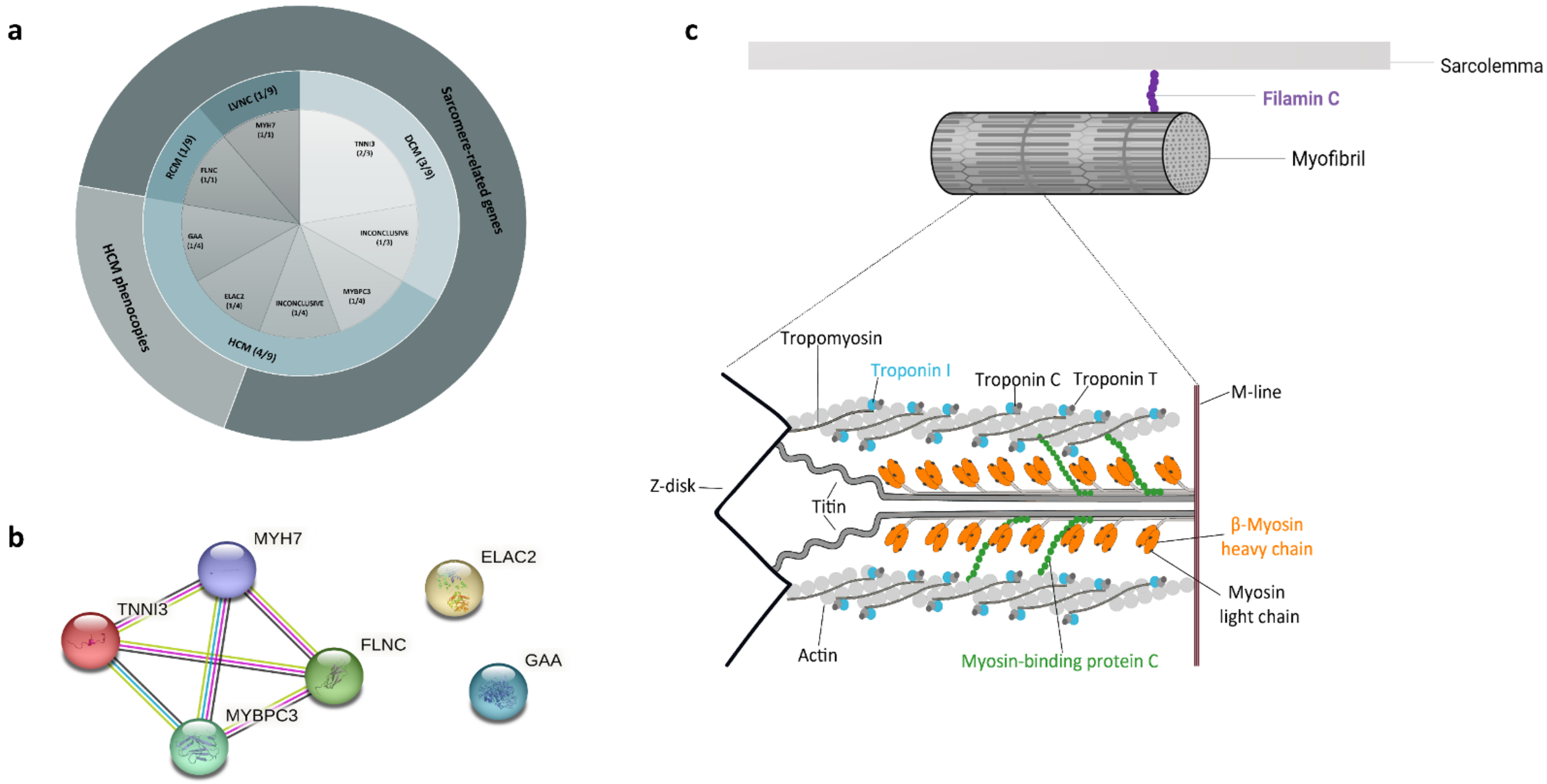

Fig S1.Representation of the main findings in our study. a. Distribution of sarcomere-related genes and genes underlying HCM phenocopies in our pediatric CMP cohort. b. Protein-protein interaction network for genes linked to pediatric CMP in this study, as based on the STRING database. The network consists of nodes representing proteins and lines connecting them to show predicted functional associations. Blue line: from curated databases. Pink line: experimentally determined. Green line: textmining. Black line: co-expression. c. Illustration of sarcomere structure and attachment to sarcolemma in the cardiac muscle fiber. The main structural proteins with candidate variants discovered our CMP patients are in colors. The lower section of B is a recolored version of an image created by Mohamed Elshennawy, M.D., CC BY-SA 4.0, via Wikimedia Commons. https://commons.wikimedia.org/wiki/File:Cardiac_sarcomere_structure.png

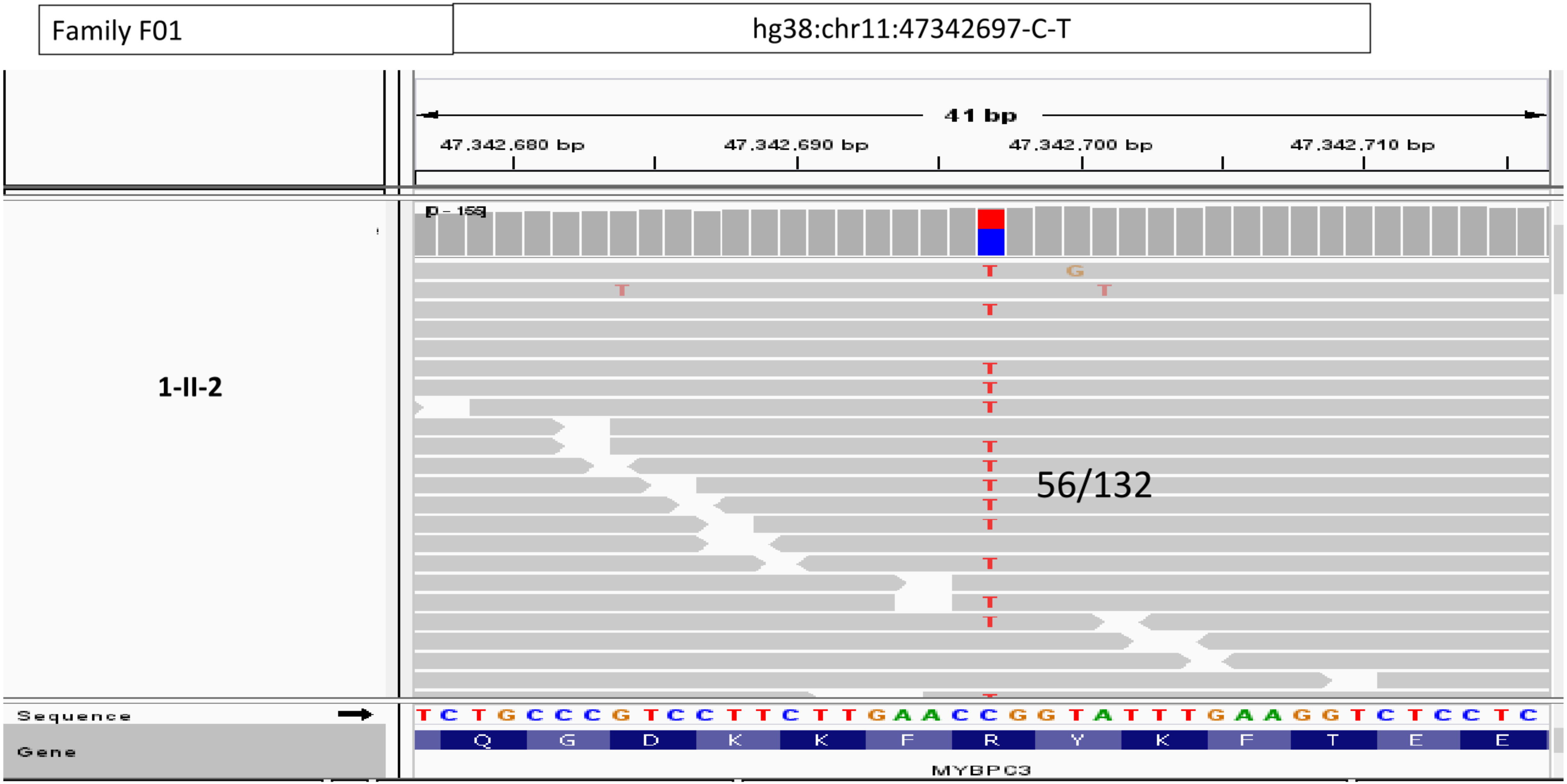

Fig S2. IGV pileups of the heterozygous NM_000256.3(*MYBPC3*):c.1505G>A variant in 1-II-2

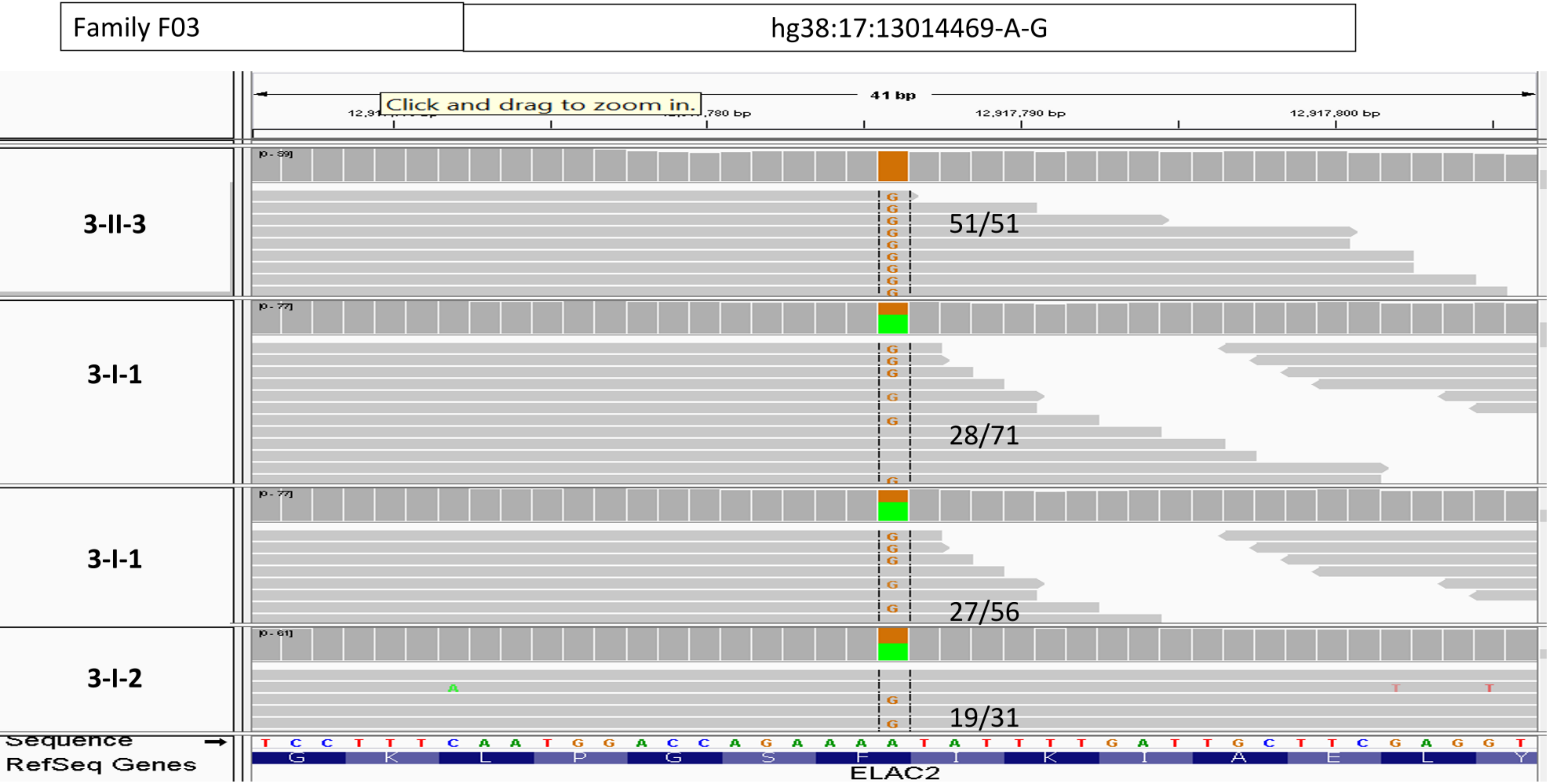

Fig S3. IGV pileups of the homozygous NM_018127.7(*ELAC2*):c.460T>C variant in proband 3-II-3. This variant was detected in the heterozygous form in the proband's parents.

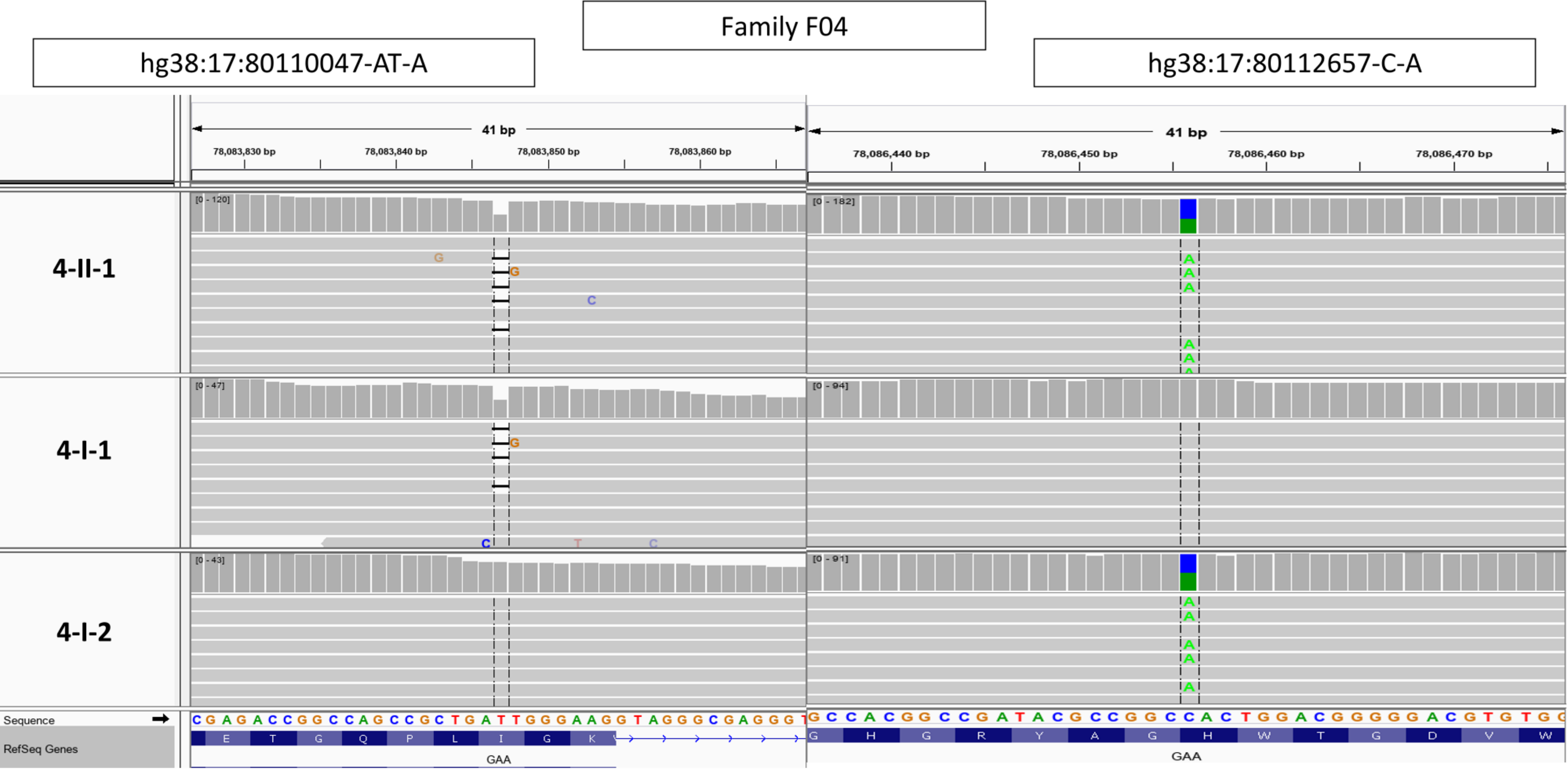

Fig S4. IGV pileups of the compound heterozygous variants NM_018127.7 (*GAA*):c.1431delT and c.1834C>A in proband 4-II-1.

Family F05	hg38:chr19:55156278-GC-G

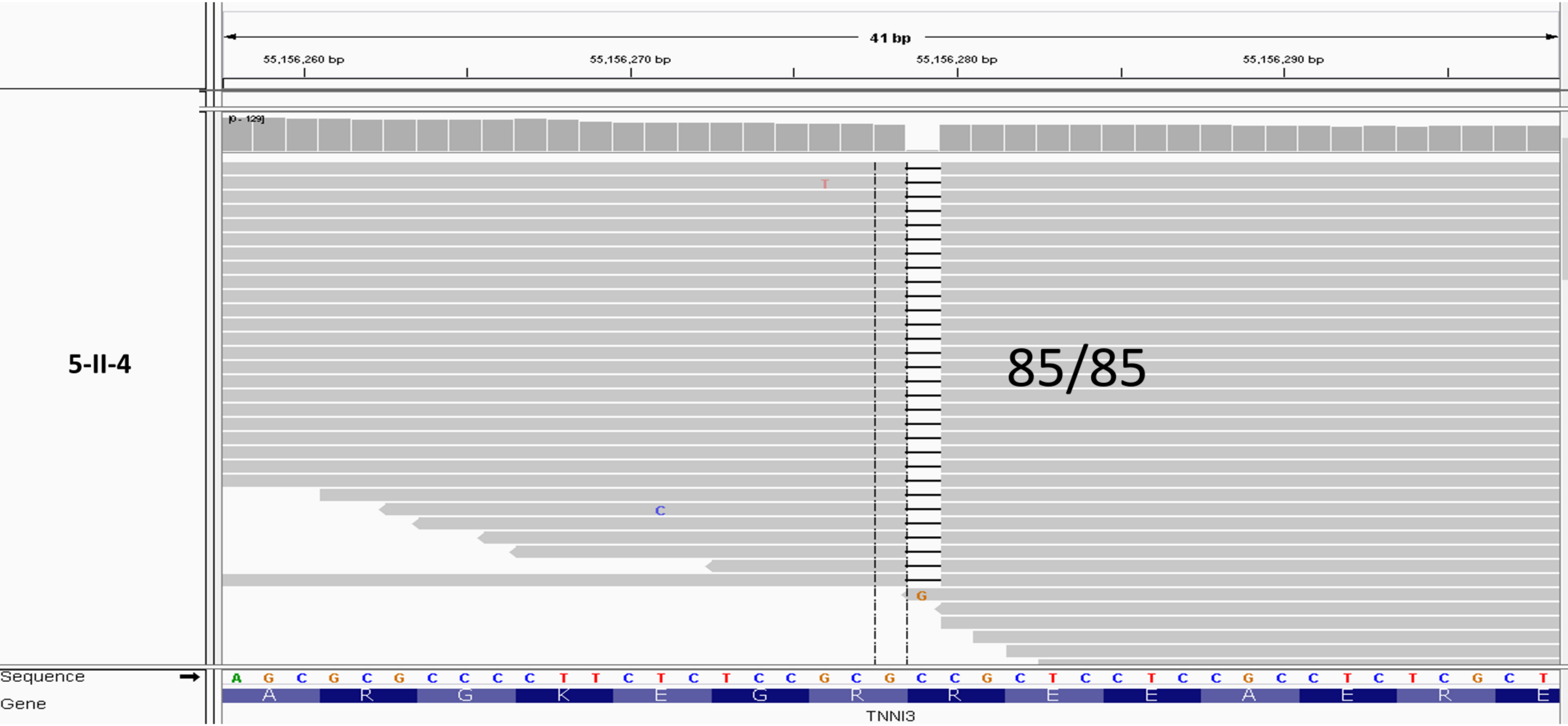

Fig S5. IGV pileups of the homozygous NM_000363.5(*TNNI3*):c.204delG variant in 5-II-4

Family F06	hg38:chr19:55156278-GC-G

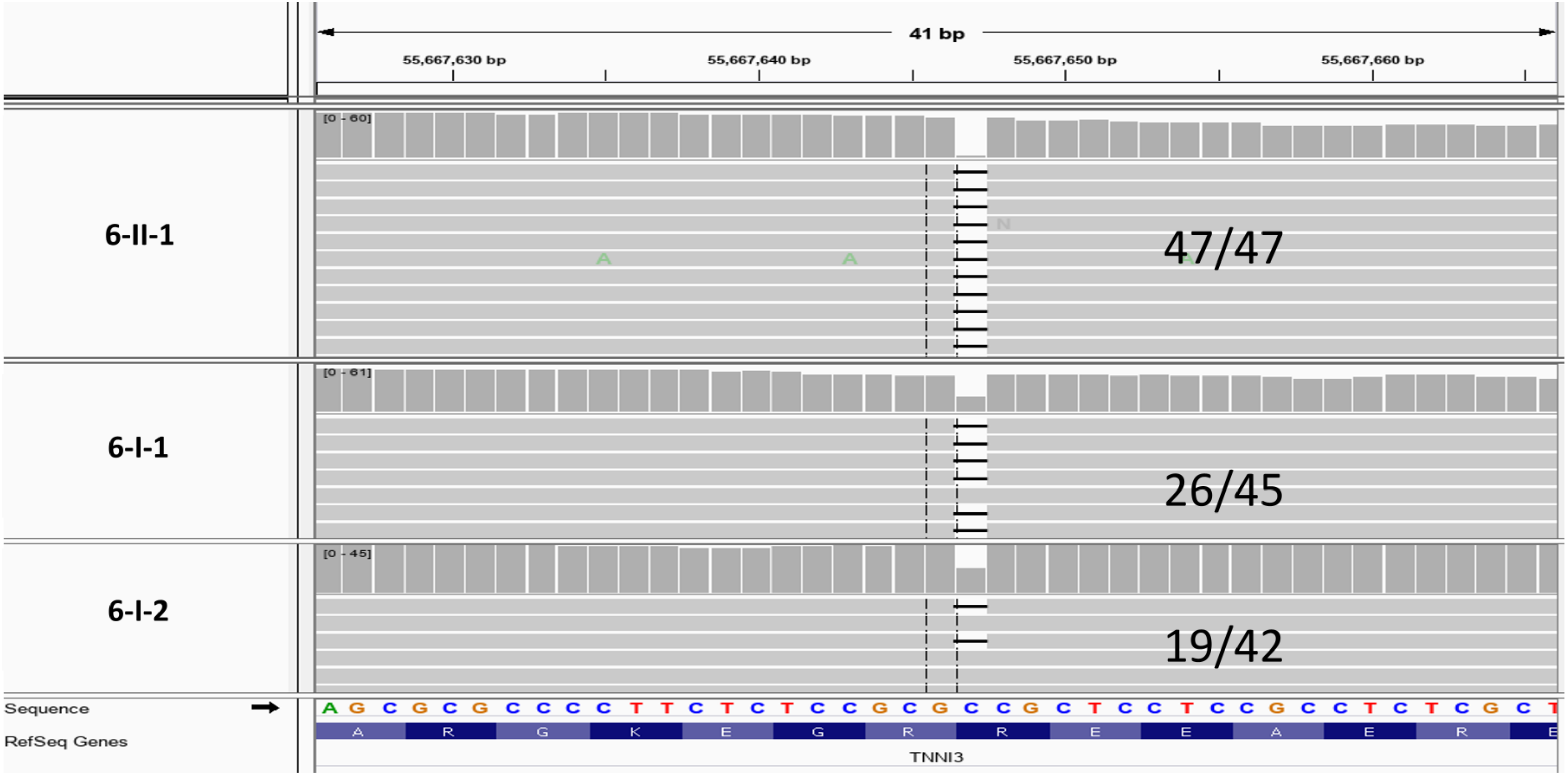

Fig S6. IGV pileups of the homozygous NM_000363.5(*TNNI3*):c.204delG variant in 6-II-1, while the variant was identified in the heterozygous state in the proband's parents

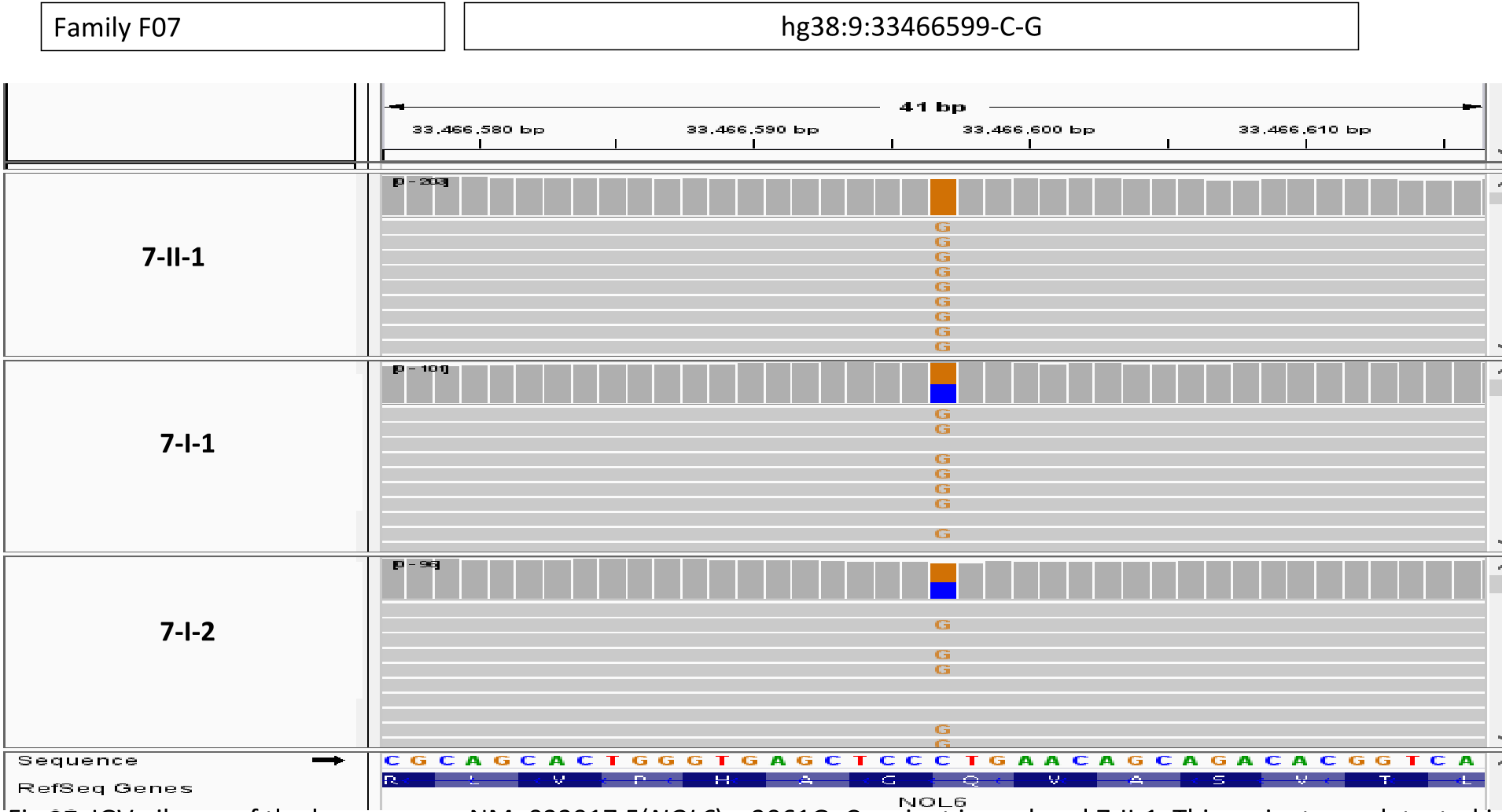

Fig S7. IGV pileups of the homozygous NM_022917.5(*NOL6*):c.2061G>C variant in proband 7-II-1. This variant was detected in the heterozygous form in the proband's parents.

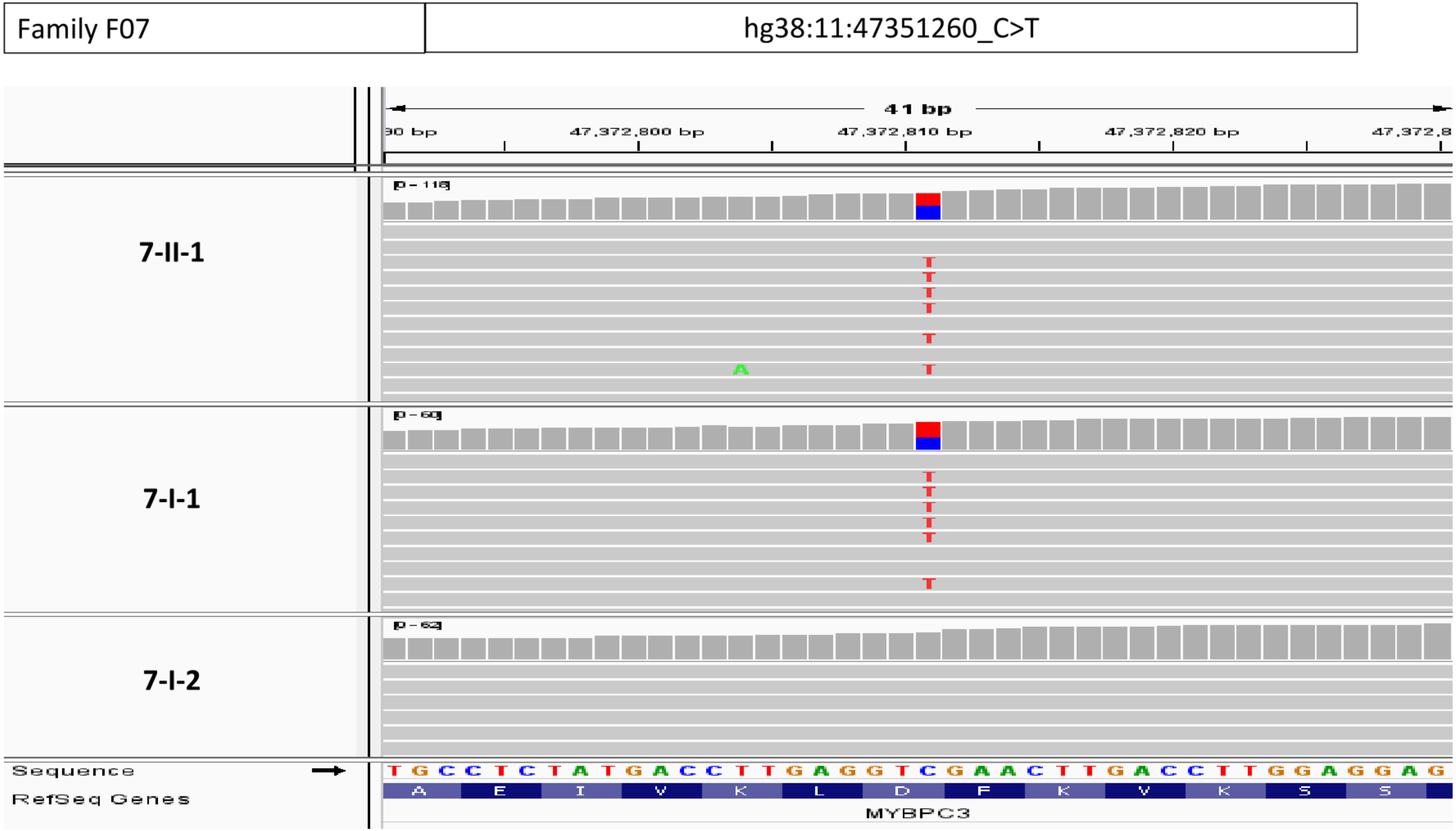

Fig S8. IGV pileups of the paternally inherited heterozygous NM_000256.3(*MYBPC3*):c.271G>A variant in proband 7-II-1.

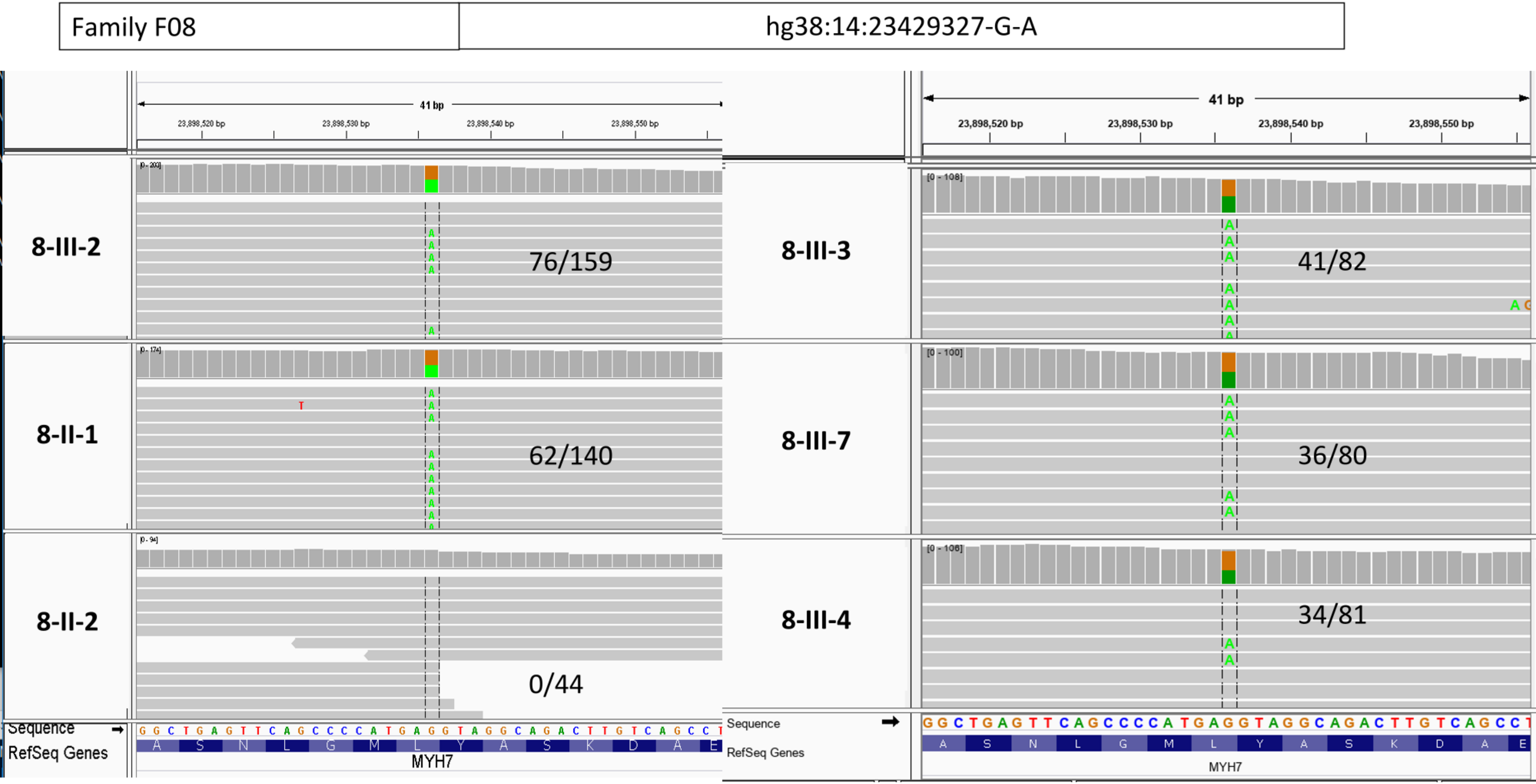

Fig S9. IGV pileups of the heterozygous NM_000257.4(*MYH7*):c.1159C>T variant in selected family members from family F08

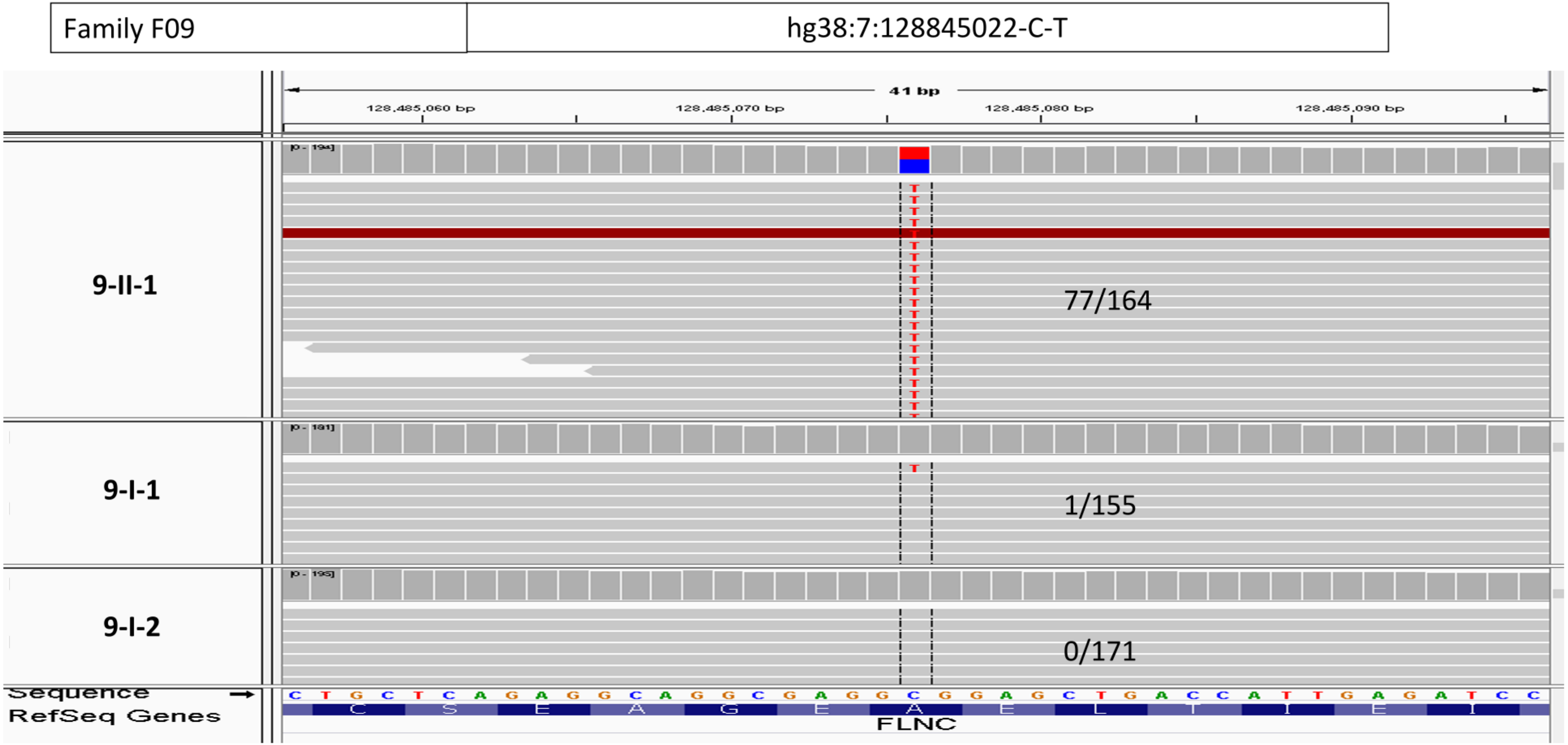

Fig S10. IGV pileups of the heterozygous NM_001458.5(*FLNC*):c.3557C>T variant in proband 9-II-1. This variant was not detected in the heterozygous form in the proband's parents.

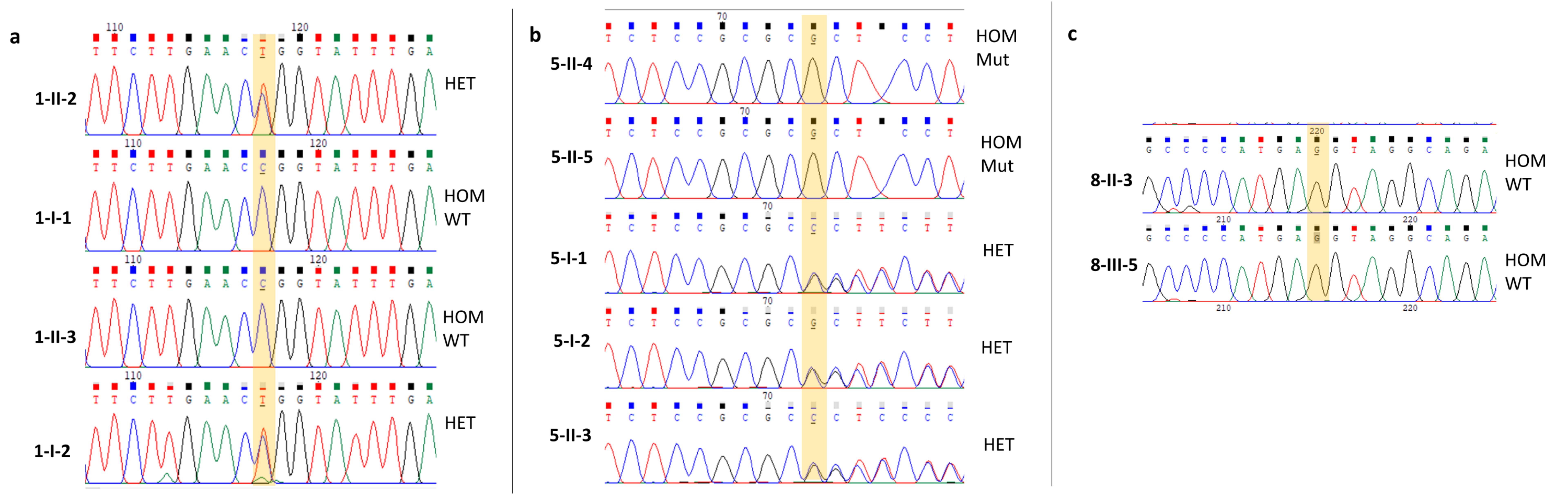

Fig S11. Sanger chromatograms of the region flanking of the detected variants in selected members from family F01 and F05. **a** visualization of the NM_000256.3(*MYBPC3*):c.1505G>A variant in family F01. **b** visualization of the NM_000363.5 (*TNNI3*):c.204delG variant in family F05. **c** visualization of the NM_000257.4 (*MYH7*):c.1159C>T variant in family F08. HET, heterozygous; HOM, homozygous; WT, wile type; Mut, mutant.
