# Supplementary material for: Unraveling the genetic tapestry of pediatric sarcomeric cardiomyopathies and masquerading phenocopies in Jordan
Source: Sci Rep. 2024 Jul 2;14:15141. doi: 10.1038/s41598-024-64921-9 (PMC11219879; doi:10.1038/s41598-024-64921-9)
Supplement: Supplementary file 3 — Supplementary Information 3. [file 41598_2024_64921_MOESM3_ESM.docx]

The first candidate variant was identified in *MYBPC3*. We identified a heterozygous paternally inherited (*MYBPC3*:c.271G>A;p.Asp91Asn) variant that changes Guanine to Adenine at position c.271 in exon 2 of the NM_000256.3 transcript (Table 2). Consequently, this change leads to the replacement of Aspartate at codon 91 of the translated MYBPC3 with Asparagine, thereby changing a polar and acidic amino acid to a polar and neutral amino acid, respectively. The *MYBPC3*:c.271G>A variant is rare in gnomADv4,indicating that it is not commonly presented in the general population.

The variant *MYBPC3*:p.Asp91Asn lacks any documented occurrence in patients with cardiomyopathies in the literature. Three different laboratories classified *MYBPC3*:p.Asp91Asn as a VUS in ClinVar. Taken together, the available data about *MYBPC3*:p.Asp91Asn is insufficient to assess the contribution of this variant to the patient’s phenotype. Therefore, we classify *MYBPC3*:p.Asp91Asn as a variant of uncertain significance (VUS).

We identified a candidate variant in the homozygous form in the *ADGRL2* gene. This *ADGRL2*:c.61C>A variant in exon 2 leads to the substitution of a proline residue at amino acid position 21 of the NM_001366006.2 transcript with threonine (p.Pro21Thr). According to the gnomAD v4 database, this variant has a maximum minor allele frequency (MAF) of 0.000007740 in the East Asian population and MAF of 0.0001678 in Middle Easten. To date, the *ADGRL2*:p.Pro21Thr variant has not been reported in ClinVar nor in individuals with relevant clinical manifestations. The observed substitution in *ADGRL2* has been classified as a VUS based on the limited available evidence.

*ADGRL2* belongs to a subfamily of G-protein coupled receptors, called latrophilin. This gene is also known as latrophilin-2 (*LPHN2*) [1]. Currently, no OMIM entry is available for *ADGRL2*. An in vitro differentiation of mouse pluripotent stem cells (PSCs) identified *ADGRL2* has as a cell-surface marker for cardiomyogenic lineage [2]. The work by Lee et al., on Lphn2-Knock out (KO) embryonic cells and *Lphn2*-KO mice model showed that Lphn2 is necessary for the expression of key cardiac-related genes, such as *Nkx2.5*, *Gata4* and *Tbx5*. The homozygous *Lphn2* (*Lphn2*^−/−^) mice model resulted in embryonic lethality and revealed major malformations in the cardiac outflow tract, right atrium and right ventricle. Furthermore, the left ventricle’s muscle mass was significantly reduced in size in comparison with the heterozygous (*Lphn*2^+/-^) and wild-type embryos [2]. Furthermore, variants in *ADGRL2* have been reported in patients with autism spectrum disorder and schizophrenia [3],[4]. *ADGRL2* is a gene of uncertain significance, as its specific phenotypic implications are not well defined to date. Further investigations are needed to study the role of *ADGRL2* in human cardiac development and its potential involvement in neurodevelopmental disorders.

The third candidate variant was found in the homozygous form in the *NOL6* gene. The variant *NOL6*:c.2061G>C in exon 16 changes a glutamine residue at amino acid 687 of the NM_022917.5 transcript to histidine (p.Gln687His). The *NOL6*:p.Gln687His leads to the substitution of a polar and neutral amino acid with a polar and basic amino acid. Based on gnomADv4 database, this variant has a maximum MAF of 0.0008248 in the Middle Eastern population. The NOL6:p.Gln687His variant has neither been previously reported in ClinVar nor in patients with cardiac defects.

*NOL6* interacts with a gene that has a provisional link to causing autosomal recessive primary microcephaly type 28, called *RRP7A* (OMIM:619453) [5]. At present, *NOL6* is considered a gene of uncertain significance (GUS) with unclear phenotypic implications. Interestingly, two LoF variants in *NOL6* have been associated with causing autism spectrum disorder [6]. Proband 7-II-1 has been described as showing autism spectrum disorder. Based on the limited evidence about the clinical implications of NOL6:p.Gln687His, we classify this variant as a VUS.

The fourth candidate variant was found in *PIGQ*. We identified a homozygous missense variant (c.831C>A) in the transcript NM_004204.5. The variant *PIGQ*:c.831C>A will lead to the substation of p.Asn277 to Lys. The *PIGQ*:c.831C>A has not been found in the population database (gnomADv4), ClinVar, or literature.

Biallelic variants in *PIGQ* have been associated with causing multiple congenital anomalies-hypotonia-seizures syndrome type 4 (OMIM: 618548) [7]. Patients with pathogenic variants in *PIGQ* have been reported to manifest global developmental delay, cardiac manifestations, encompassing arrythmia, heat block, and pulmonary stenosis with variable expressivity [7],[8]. Taken together, the current evidence about *PIGQ*:c.831C>A is insufficient to conclude its pathogenicity or its implication in causing Proband’s 7-II-1 clinical picture. Therefore, we sought to classify *PIGQ*:c.831C>A as a VUS.

The current state of knowledge to implicate any of these variants to be causing Proband’s 7-II-1 clinical manifestation is currently limited and cannot be concluded based on the present level of evidence.

References:

1. Lee, C. S. *et al.* Adhesion GPCR Latrophilin-2 Specifies Cardiac Lineage Commitment through CDK5, Src, and P38MAPK. *Stem Cell Reports* **16**, 868–882 (2021).

2. Lee, C. S. *et al.* Identification of Latrophilin-2 as a Novel Cell-Surface Marker for the Cardiomyogenic Lineage and Its Functional Significance in Heart Development. *Circulation* **139**, 2910–2912 (2019).

3. Fromer, M. *et al.* De novo mutations in schizophrenia implicate synaptic networks. *Nature* **506**, 179–184 (2014).

4. Li, J. *et al.* Genes with de novo mutations are shared by four neuropsychiatric disorders discovered from NPdenovo database. *Mol. Psychiatry* **21**, 290–297 (2016).

5. Farooq, M. *et al.* RRP7A links primary microcephaly to dysfunction of ribosome biogenesis, resorption of primary cilia, and neurogenesis. *Nat. Commun.* **11**, (2020).

6. Kosmicki, J. A. *et al.* Refining the role of de novo protein-truncating variants in neurodevelopmental disorders by using population reference samples. *Nat. Genet.* **49**, 504–510 (2017).

7. Johnstone, D. L. *et al.* Early infantile epileptic encephalopathy due to biallelic pathogenic variants in PIGQ: Report of seven new subjects and review of the literature. *J. Inherit. Metab. Dis.* **43**, 1321–1332 (2020).

8. Starr, L. J., Spranger, J. W., Rao, V. K., Lutz, R. & Yetman, A. T. PIGQ glycosylphosphatidylinositol-anchored protein deficiency: Characterizing the phenotype. *Am. J. Med. Genet. A* **179**, 1270–1275 (2019).
